# Circadian disruption and ROS-NLRP3 signaling mediate sleep deprivation-enhanced silica nanoparticle toxicity in lacrimal glands

**DOI:** 10.1186/s12951-025-03630-5

**Published:** 2025-09-02

**Authors:** Wenxiao Zhang, Di Qi, Xiaoting Pei, Dingli Lu, Mengru Ba, Shuting Xuan, Duliurui Huang, Tingting Yang, Jingwen Yang, Zhijie Li, Shenzhen Huang

**Affiliations:** 1 Department of Ophthalmology, People’s Hospital of Zhengzhou University, Henan Provincial People’s Hospital, Zhengzhou, China; 2 Henan Key Laboratory of Ophthalmology and Visual Science, Henan Eye Hospital and Henan Eye Institute, People’s Hospital of Henan University, People’s Hospital of Zhengzhou University, Henan Provincial People’s Hospital, Zhengzhou, China; 3 Department of Ophthalmology, People’s Hospital of Henan University, Henan Provincial People’s Hospital, Zhengzhou, China; 4 Henan Key Laboratory of Ophthalmology and Visual Science, Henan Eye Hospital and Henan Eye Institute, Henan Provincial People’s Hospital, No. 7, Weiwu Road, Zhengzhou, 450003 China

**Keywords:** Circadian Rhythm Disruption, Lacrimal Gland Dysfunction, Oxidative Stress, NLRP3 Inflammasome, Environmental Nanotoxicology

## Abstract

Sleep deprivation (SD) and exposure to engineered nanomaterials such as silica nanoparticles (SiNPs) are emerging risk factors for ocular surface disorders, particularly dry eye disease. However, the molecular mechanisms underlying their combined impact on lacrimal gland function remain unclear. In this study, we investigated the synergistic effects of SD and SiNPs exposure on circadian regulation, oxidative stress, inflammation, and structural integrity of the extraorbital lacrimal glands (ELGs) in C57BL/6J mice. Behavioral and physiological monitoring revealed that SD + SiNPs disrupted circadian locomotor activity and body temperature rhythms. Phenotypic assessments showed reduced tear secretion and ELG atrophy. RNA sequencing identified extensive transcriptomic reprogramming, including altered expression of core clock genes and enrichment of inflammatory and redox-related pathways. Increased reactive oxygen species (ROS) accumulation and γ-H2AX expression indicated oxidative DNA damage. Immunohistochemistry confirmed NLRP3 inflammasome activation, while Western blotting revealed enhanced phosphorylation of JAK2, STAT3, NF-κB p65, and IκBα, alongside upregulation of IL-17A. Elevated malondialdehyde levels further reflected oxidative lipid damage. These findings demonstrate that SD exacerbates SiNPs-induced ELG dysfunction via circadian disruption and activation of the ROS/NLRP3/IL-17A inflammatory axis. While these effects are currently limited to the lacrimal gland, future studies are needed to determine whether similar mechanisms contribute to broader systemic metabolic consequences.

**Graphical abstract:**

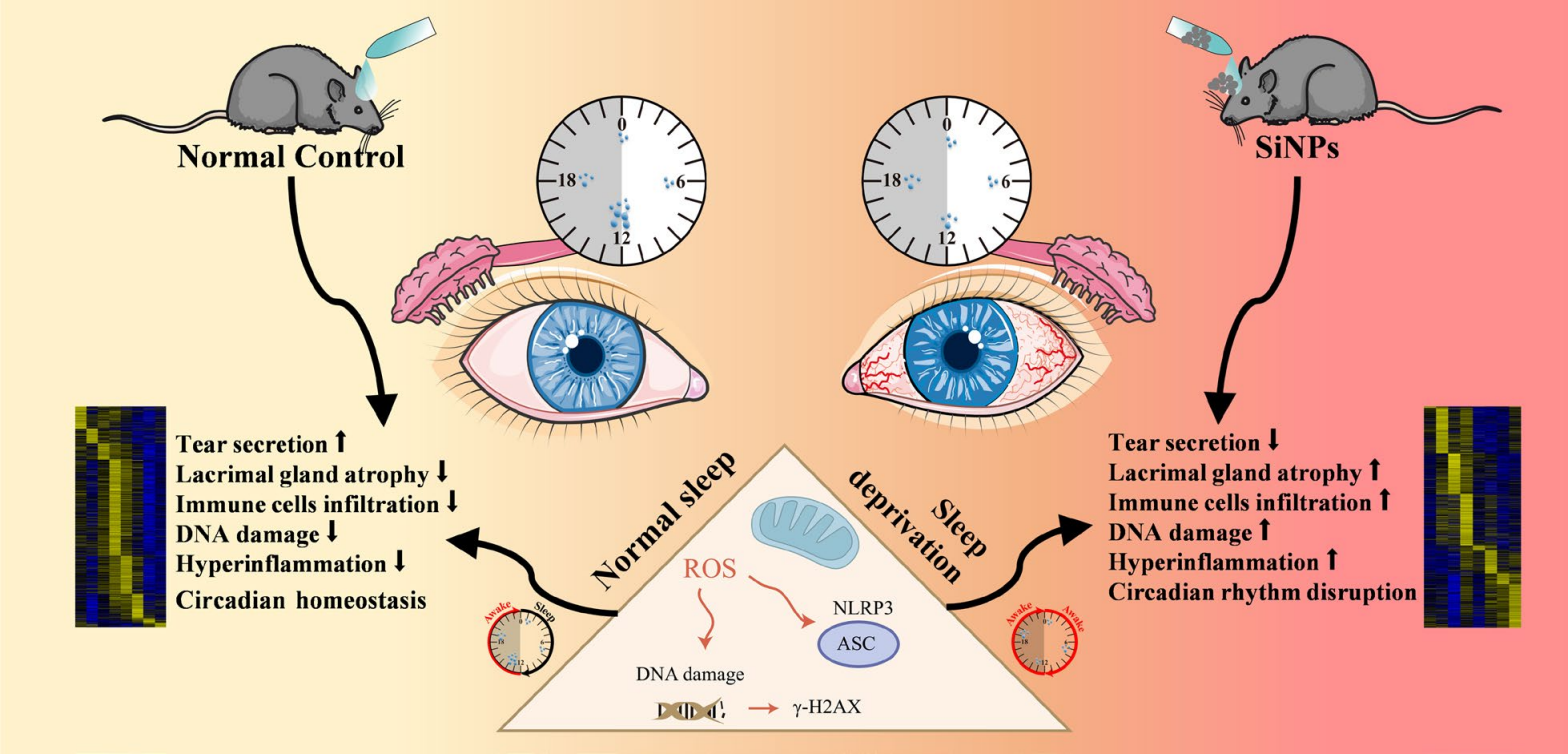

**Supplementary Information:**

The online version contains supplementary material available at 10.1186/s12951-025-03630-5.

## 1. Background

Nanoparticles possess unique physicochemical properties that make them highly versatile across diverse fields such as drug delivery, electronics, and cosmetics [1–3]. Among these, silica nanoparticles (SiNPs) have received particular attention due to their tunable size, favorable biocompatibility, and modifiable surfaces [4]. Despite their broad utility, concerns about their potential toxicity are mounting [5–7]. SiNPs can traverse biological barriers and exert cytotoxic effects on critical organs including the brain, liver, and kidneys [8, 9]. Recently, their potential to damage ocular tissues—especially the lacrimal glands—has emerged as a pressing issue. However, the systemic and ocular safety of SiNPs, particularly regarding their impact on lacrimal gland integrity, remains poorly understood.

Sleep deprivation (SD) is a growing global health concern, associated with a range of systemic disorders such as diabetes, hypertension, and cardiovascular disease [10, 11]. Beyond these systemic effects, SD is increasingly recognized as a contributor to ocular surface disorders, particularly dry eye disease [12–14]. SD has been shown to disrupt circadian regulation and impair tear secretion, leading to structural and functional changes in murine extraorbital lacrimal glands (ELGs) [15]. The co-existence of SD and nanoparticle exposure may synergistically aggravate ELG damage, posing a compounded risk to ocular health. However, the underlying molecular mechanisms remain inadequately elucidated.

Reactive oxygen species (ROS), as critical mediators of oxidative stress, are increasingly implicated in ocular surface pathologies, including lacrimal gland dysfunction and dry eye disease [16, 17]. Under physiological conditions, ROS generation is tightly regulated by endogenous antioxidant systems; however, excessive ROS accumulation disrupts redox homeostasis, triggering lipid peroxidation, DNA damage, and inflammatory cascades [18, 19]. The NOD-, LRR- and pyrin domain-containing protein 3 (NLRP3) inflammasome, a multiprotein complex activated by cellular stress signals, serves as a molecular nexus linking ROS overproduction to inflammatory tissue injury [20, 21]. Mechanistically, ROS facilitate NLRP3 inflammasome assembly by promoting mitochondrial dysfunction, potassium efflux, and lysosomal destabilization [22], culminating in caspase-1 activation and subsequent cleavage of pro-inflammatory cytokines [23]. Emerging evidence suggests that nanoparticle exposure amplifies this pathway through sustained oxidative burst and inflammasome priming [21, 24], while SD exacerbates ROS generation via circadian disruption of antioxidant enzymes [25]. Notably, lacrimal glands exhibit heightened vulnerability to ROS/NLRP3-mediated damage due to their high metabolic activity and exposure to airborne particulates [16, 26]. We hypothesize that SD and SiNPs synergistically disrupt redox balance and circadian homeostasis, creating a pathological loop of ROS accumulation and NLRP3-driven inflammation [25, 27, 28]. However, whether this pathway mediates SiNPs-induced lacrimal gland injury remains to be determined.

To address these challenges, we established a mouse model to investigate the combined effects of SD and SiNPs on the ELG function. We focused on the ROS/NLRP3 signaling axis to explore its contribution to inflammation, oxidative stress, and circadian dysregulation. This study aims to provide mechanistic insights into the co-pathogenic effects of behavioral and environmental stressors, potentially guiding the development of targeted therapies for lacrimal gland dysfunction and dry eye disease.

## 2. Materials and methods

### 2.1 Overall study scheme and analysis workflow

**Fig. 1:**
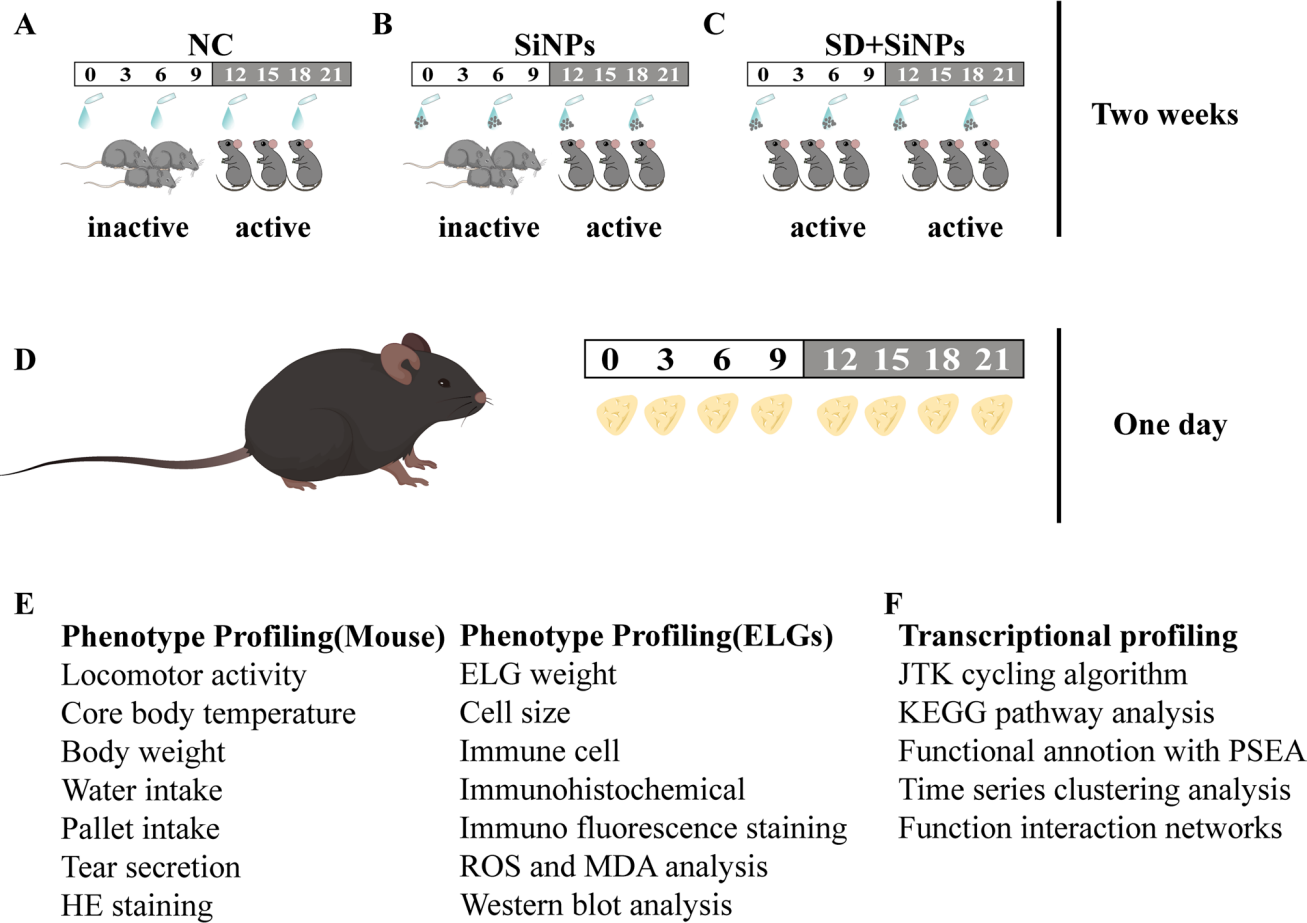
Experimental design and analysis workflow for studying of SiNPs and the combined effects of SD and SiNPs. (**A**-**C**) Schematic representation of the behavioral activity monitoring protocol over a two-week period for the NC, SiNPs-treated, and SD + SiNPs-treated groups. The transition from the inactive phase (gray) to the active phase (black) is indicated. **(D)** The schematic diagram illustrates the experimental timeline for ELG collection over a 24-hour period. **(E)** Overview of phenotype profiling and analysis. The assessed phenotypes included locomotor activity, core body temperature, body weight, water intake, pellet intake, and tear secretion. ELG analysis involved measurements of ELG weight, cell size, immune cell infiltration, immunohistochemical analysis, ROS, and MDA analysis, and Western blot analysis. **(F)** Transcriptional profiling of ELGs was conducted, including the JTK cycling algorithm, KEGG pathway analysis, functional annotation using PSEA, time-series clustering analysis, and functional interaction network construction

The experimental framework of this study is summarized in Fig. 1. We aimed to evaluate the effects of SiNPs on the ELGs and to investigate the potential synergistic impact of combined SiNPs exposure and SD (SD + SiNPs). Male C57BL/6J mice were used for all experiments. The SD model was established using a dedicated apparatus capable of inducing mechanical arousal and concurrently delivering ocular SiNPs exposure, as illustrated in Fig. 1. A separate SiNPs-only exposure model was implemented following previously described protocols [29–31].

After two weeks of treatment, body weight was recorded, and water and pellet consumption were carefully monitored. Locomotor activity and core body temperature were assessed using standard telemetry-based methods [32, 33]. Mice were divided into three groups: normal control (NC), SiNPs-treated, and SD + SiNPs-treated. ELG samples were collected from each group at three-hour intervals over a 24-hour period (*n* = 3 per time point), enabling temporal analysis **(**Figs. 1A-D**)**.

Collected ELGs were subjected to RNA sequencing (RNA-Seq), histological, Western blot, or immunohistochemical analyses. Transcriptomic profiling was conducted using a comprehensive bioinformatics workflow. This included identification of rhythmic genes via the Jonckheere-Terpstra-Kendall (JTK_CYCLE) algorithm, Kyoto Encyclopedia of Genes and Genomes (KEGG) pathway analysis, and construction of functional interaction networks. Additionally, time-series clustering analysis and phase set enrichment analysis (PSEA) were applied to elucidate molecular alterations induced by SiNPs exposure alone or in combination with SD **(**Figs. 1E-F**)**.

### 2.2 Animals and models

Male C57BL/6J mice (10–12 weeks old) were obtained from Gempharmatech Co., Ltd (Nanjing, Jiangsu, China). All experimental procedures involving animals were conducted in compliance with the guideline set by the ARVO Statement for the Use of Animals in Ophthalmic and Vision Research, and were approved by the Henan Province People’s Hospital Institutional Animal Care and Use Committee (HNEECA-2024-08). Mice were housed under controlled environmental conditions with a 12:12-hour light/dark (LD) cycle (lights on at 7:00 a.m., off at 7:00 p.m.), constant temperature (23–25 °C), relative humidity (50–70%), and light intensity (15–20 lx), with *ad libitum* access to food and water [34, 35].

The experimental design included three groups: (1) a normal control (NC) group receiving no treatment, (2) a SiNPs-treated group receiving topical nanoparticle exposure only, and (3) a combined SD + SiNPs group subjected to both SD and nanoparticle treatment. All groups were maintained in light-tight circadian chambers under the same LD cycle [36].

Topical ocular exposure to SiNPs was performed according to a previously described irritation test protocol [31]. Briefly, 0.4% oxybuprocaine hydrochloride was instilled into the eyes to induce local anesthesia. After 5 min, the lower eyelid was gently retracted and 2 µL of a SiNPs suspension (1 mg/mL in deionized water) was applied to the conjunctival sac. The eyelids were held closed for 1 s to prevent loss of the solution. The SiNPs used were spherical, 30 ± 5 nm in diameter (Cat. No. S817575, Shanghai Macklin Biochemical Technology Co., Ltd., China). Eye drops were prepared by diluting SiNPs to 5 mg/mL in deionized water, followed by vortexing and ultrasonication, and stored at 4 °C [37].

Mice in the NC group received identical handling and deionized water as vehicle control. SiNPs treatments were administered topically four times daily for 14 consecutive days. In the SD + SiNPs group, SD was induced using a custom-built device that activated a sweep bar every 1.5 min during the 12-hour light phase (zeitgeber time [ZT] 0–12) [25, 38–40]. SiNPs eye drops were administered at ZT0, ZT6, ZT12, and ZT18 over the two-week period. At the end of the treatment, ELGs were collected at three-hour intervals across 24 h from all groups for downstream analyses.

### 2.3 Tissue collection and RNA extraction

ELG tissues were collected from mice in the NC, SiNPs-treated, and SD + SiNPs-treated groups at eight circadian time points: ZT0, ZT3, ZT6, ZT9, ZT12, ZT15, ZT18, and ZT21, with three biological replicates per group at each time point (*n* = 3) [33, 36, 41, 42]. Immediately after dissection, ELG samples were snap-frozen in liquid nitrogen and stored at −80 °C to preserve RNA integrity.

Total RNA was extracted using the RNeasy Mini Kit (Qiagen, Hilden, Germany) according to the manufacturer’s protocol. RNA concentration and purity were assessed using a NanoDrop 2000c spectrophotometer (Thermo Fisher Scientific, Waltham, MA, USA).

### 2.4 RNA-Seq

A total of 72 ELG samples collected across the specified ZT points underwent RNA purification for transcriptomic analysis. At each time point, triplicate samples were prepared, with each sample consisting of pooled left and right ELG tissues from a single mouse. RNA-Seq and subsequent data analysis were performed on the BGIseq500 platform (BGI-Shenzhen, Guangzhou, China) according to standard protocols [36, 41]. This process included mRNA fragmentation, cDNA library construction, and quality assessment using an Agilent Technologies 2100 bioanalyzer. Sequencing was conducted using probe-anchored polymerization technology. Gene expression in the NC, SiNPs, and SD + SiNPs-treated samples was normalized using fragments per kilobase of transcript per million mapped reads (FPKM) values, with three mice per group per ZT.

### 2.5 Detection of rhythmic gene expression

To identify genes with circadian rhythmicity in ELG samples, the JTK_CYCLE algorithm implemented in the R statistical environment was applied, following previously established protocols [43]. The analysis was based on normalized FPKM values across eight circadian time points. Genes were categorized into three groups—rhythmic, non-rhythmic, and low-expressed—based on their temporal expression patterns and adjusted *P*-values. This classification enabled the evaluation of how SiNPs exposure and SD treatment influence circadian gene expression in ELGs. Each time point included three biological replicates per group (*n* = 3 per ZT).

### 2.6 Functional annotation with KEGG and PSEA

KEGG pathway enrichment analysis was performed by comparing our data against the KEGG V81.0 database and the NCBI RefSeq (GCF_000001635.25_GRCm38.p5), as previously described [44]. We determined significantly enriched pathways based on *P*-values < 0.01. Temporal clustering pathways were identified using PSEA software (version 1.1), with a cutoff of *Q* < 0.05, as previously described [43]. For this purpose, the C2 (KEGG gene sets) dataset labeled “c2.cp.kegg.v6.2.symbols.gmt” from the Molecular Signatures Database (MSigDB) was utilized. The Kuiper statistic was used to identify significantly enriched pathways in the ELGs of NC, SiNPs-treated, and SD + SiNPs-treated groups, with *Q*-value < 0.05 cutoff applied. This analysis involved a total of 24 individual mice per group.

### 2.7 Time-series clustering analysis

The Mfuzz soft clustering tool was used to identify distinct clusters within the circadian gene datasets, as previously described [45]. FPKM values were clustered according to parameters specified in Mfuzz. The resulting cluster structures were visualized using data from three mice per group at various ZTs.

### 2.8 Tear secretion measurement

Aqueous tear secretion volume was quantified using phenol red thread (Jingming, Tianjin, China) as previously reported [36, 46]. Briefly, a phenol red-dyed cotton thread was placed on the palpebral conjunctiva approximately one-third of the distance from the lateral canthus of the eyelid for 20 s. The lengths of the tear-wetted threads were then measured in millimeters. To evaluate diurnal variation in tear production, measurements were taken at four ZT points—ZT0, ZT6, ZT12, and ZT18—on experimental Day 30. At each time point, six mice were assessed per group (NC, SiNPs-treated, and SD + SiNPs-treated), totaling 24 mice per group. Time points were selected based on previously published studies on ocular circadian physiology [33, 36, 41, 42, 47].

### 2.9 Hematoxylin and eosin staining and ELG immunohistochemistry

For histological and eosin (HE) and immunohistochemical analysis, ELGs were harvested at four circadian time points (ZT0, ZT6, ZT12, ZT18) from six mice per time point per group, totaling 24 mice per group (NC, SiNPs-treated, and SD + SiNPs-treated). Tissues were fixed in 4% paraformaldehyde to preserve tissue integrity and structure.

HE staining was conducted according to established protocols. The staining procedure involved incubating the tissue sections in hematoxylin solution for 3–5 min, followed by a 5-minute application of eosin dye. This staining technique allows for the differentiation of cellular components, with hematoxylin staining the nuclei blue and eosin coloring the cytoplasm and extracellular matrix in pink. After staining, the sections were meticulously sealed with neutral gum to prevent degradation and to enhance the longevity of the slides. Slides were sealed with neutral gum and imaged using a NIKON ECLIPSE E100 upright microscope with a DS-U3 imaging system.

For immunohistochemistry, ELG sections of the NC, SiNPs-treated, and SD + SiNPs-treated groups were washed and blocked in 3% bovine serum albumin (Cat no. G5001, Servicebio Company, Wuhan, China) in PBS at pH 7.4 and incubated overnight at 4 °C with anti-CD4 antibody (Cat no. GB13064-2, Servicebio Company, Wuhan, China), anti-CD8 antibody (Cat no. GB13429, Servicebio Company, Wuhan, China), anti-NLRP3 antibody (Cat no. 68102-1-Ig, Proteintech, Wuhan, China), anti-ASC antibody (Cat no. ab309497, Abcam, USA), anti-γ-H2Ax antibody (Cat no. 7631 T; Cell Signaling Technology, Inc.), anti-beta III tubulin monoclonal antibody (Cat. no. GB12139; Servicebio Company, Wuhan, China). After washing, slides were incubated with HRP-conjugated secondary antibodies and imaged under brightfield or fluorescence microscopy.

### 2.10 Western blot analysis

Frozen mouse ELGs were homogenized and lysed in radioimmunoprecipitation assay (RIPA) buffer (Cat no. G2002-100ML, Servicebio Company, Wuhan, China) on ice for 30 min. Lysates were centrifuged (10 min, 4 °C) to collect supernatants. Samples (2 µg/µL) were boiled for 5 min, resolved via 10% SDS-PAGE, and electrotransferred to PVDF membranes (0.45 μm; Cat no. WGPVDF45, Servicebio Company, Wuhan, China). Membranes were blocked with 5% bovine serum albumin (BSA; Beyotime) for 1 h at 25 °C and incubated overnight at 4 °C with the following primary antibodies: IκB Alpha (Cat no. 10268-1-AP; Proteintech, Wuhan, China), Phospho-IκB Alpha (Cat no. 82349-1-RR; Proteintech, Wuhan, China), NF-κB p65 (Cat no. 10745-1-AP; Proteintech, Wuhan, China), Phospho-NF-κB p65 (Cat no. 82335-1-RR; Proteintech, Wuhan, China), JAK2 (Cat no. GB11325, Servicebio Company, Wuhan, China), Phospho-JAK2 (Cat no. GB114585, Servicebio Company, Wuhan, China), STAT3 (Cat no. GB11176, Servicebio Company, Wuhan, China), Phospho-STAT3 (Cat no. GB150001, Servicebio Company, Wuhan, China), and IL-17A (Cat no. GB11289, Servicebio Company, Wuhan, China) diluted 1:1000 in 2% BSA. After three washes with PBS containing 0.1% Tween-20 (Beyotime), membranes were incubated with HRP-conjugated secondary antibodies (1:3000 in 2% BSA, Cat no. GB23303, Servicebio Company, Wuhan, China). β-actin (Cat no. GB15003, Servicebio Company, Wuhan, China) served as the loading control.

### 2.11 ROS and MDA assays

Following euthanasia, ELGs were collected from mice in the NC, SiNPs-treated, and SD + SiNPs-treated groups. Tissues were embedded in optimal cutting temperature compound (O.C.T.; Tissue-Tek, Cat no. G6059, Servicebio Company, Wuhan, China). After rewarming to room temperature, sections underwent autofluorescence quenching (Cat no. G1221, Servicebio Company, Wuhan, China) for 5 min. Subsequent rinsing with running water lasted 10 min, followed by 30-min incubation with ROS detection dye (Cat no. D7008, Sigma-Aldrich, USA) at 37 °C in darkness. Triple 5-min washes with PBS (pH 7.4; Cat no. G0002, Servicebio Company, Wuhan, China) were performed. Nuclear counterstaining employed DAPI (Cat no. G1012, Servicebio Company, Wuhan, China) for 10 min at room temperature (RT) in darkness. Air-dried sections were mounted in anti-fade medium (Cat no. G1401, Servicebio Company, Wuhan, China). ELG imaging used fluorescence microscopy (NIKON ECLIPSE C1) coupled to a DS-U3 slide scanner (Nikon), with subsequent quantification in ImageJ (v1.42q, NIH).

Total malondialdehyde (MDA) levels, as a marker of lipid peroxidation, were measured in triplicate using a commercial ELISA kit (Quantikine, R&D Systems, Minneapolis, MN, USA), following the manufacturer’s instructions.

### 2.12 Statistics and software

GraphPad Software (GraphPad Prism 8; La Jolla, CA, USA) was used for bar, scatter, and statistical analyses. Oriana software (Version 4.01; Kovach Computing Services, Pentraeth, Wales, UK) was used for analyzing the phase, period distribution, and Rayleigh vector of the oscillating gene. The Venn Diagram Plotter (https://jvenn.toulouse.inra.fr/app/index.htm) was used to compare the numbers of rhythmic genes in different groups. Heatmaps were generated by Pheatmap scripts in R (64-bit, version 4.4.2). For non-normally distributed datasets, the non-parametric Kruskal-Wallis test was used to assess intergroup differences. Comparisons among the three groups were performed using independent samples *t*-tests, one-way ANOVA with Bonferroni correction, or two-way repeated measures ANOVA with Tukey’s post hoc test. Data are presented as mean ± standard error of the mean (SEM). *P* < 0.05 was considered statistically significant. NS indicated not statistically significant.

## 3. Results

### 3.1 Sleep deprivation exacerbates circadian and behavioral disturbances in SiNPs-treated mice

**Fig. 2:**
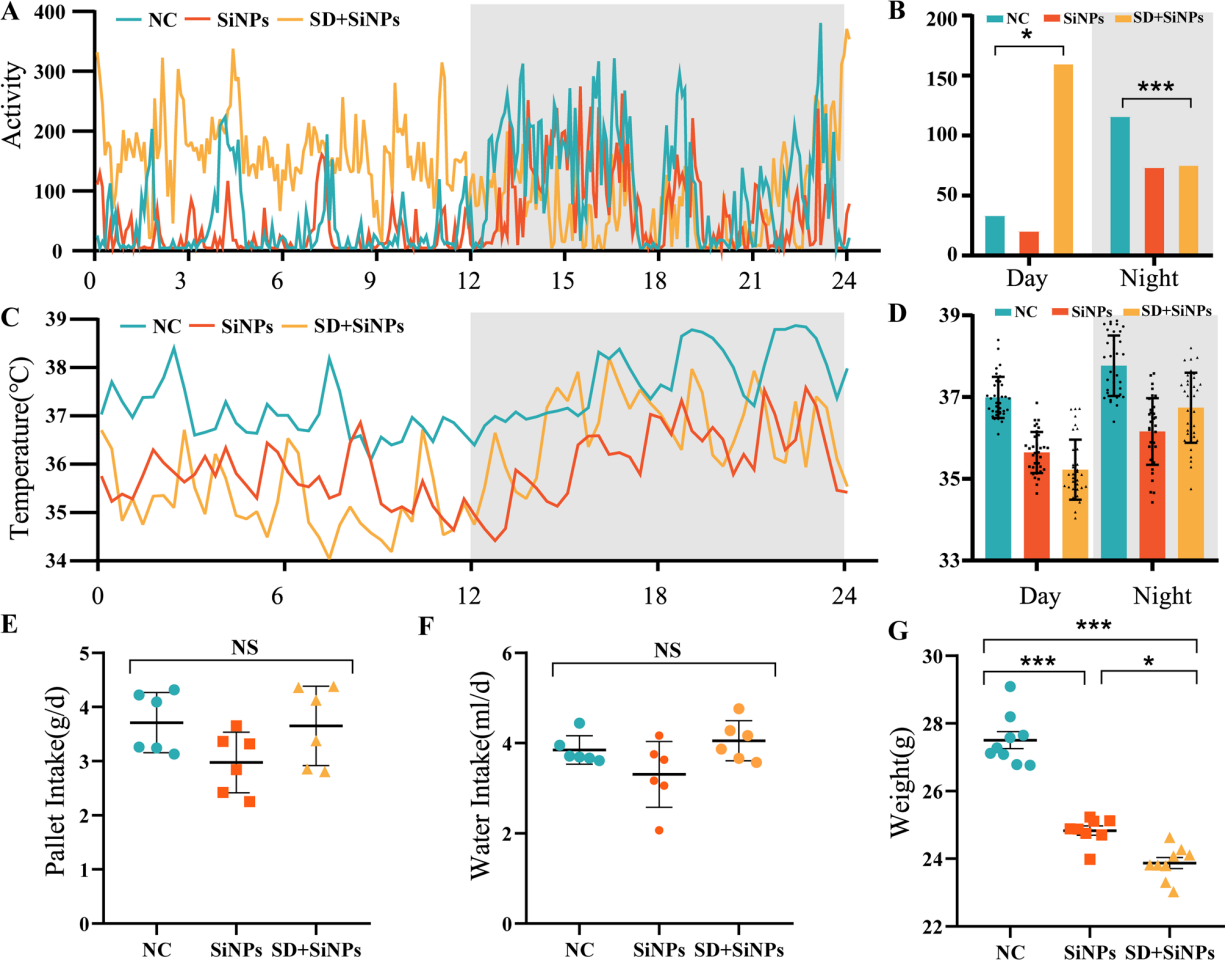
Behavioral and physiological alterations in mice following SiNPs and SD + SiNPs treatments. **(A-B)** Locomotor activity patterns over a 24-hour period in the NC, SiNPs-treated, and SD + SiNPs-treated groups. Data were collected every 5 min. The gray shading indicates the dark phase. ^*^P < 0.05, ^***^P < 0.001. **(C-D)** Core body temperature fluctuations over a 24-hour period in the NC, SiNPs-treated, and SD + SiNPs-treated groups. Data were recorded every 20 min. The gray shading indicates the dark phase. ^*^P < 0.05, ^***^P < 0.001. **(E)** Pellet consumption in the NC, SiNPs-treated, and SD + SiNPs-treated groups. NS: not significant. **(F)** Water intake in the NC, SiNPs-treated, and SD + SiNPs-treated groups. NS: not significant. **(G)** Body weight changes in the NC, SiNPs-treated, and SD + SiNPs-treated groups. ^*^P < 0.05, ^***^P < 0.001

After two weeks of treatment, pallet and water intake, body weight, core body temperature, and locomotor activity were assessed in the NC, SiNPs-treated, and SD + SiNPs-treated groups. Locomotor activity, monitored via telemetry, revealed notable alterations in the SiNPs-treated and SD + SiNPs-treated mice compared to controls **(**Figs. 2A-B**)**. During the daytime (ZT0-ZT12), the SD + SiNPs-treated group exhibited higher levels of activity than the NC and SiNPs-treated groups. Conversely, during the nighttime (ZT12-ZT24), the SD + SiNPs-treated group’s locomotor activity decreased to lower levels, while the NC and SiNPs-treated groups showed increased activity levels that were higher than those of the SD + SiNPs group. Although the SiNPs-treated group followed a similar 24-hour trend to the NC group, its average activity level over the day was lower than that of the NC group. The daily rhythms of the SiNPs-treated and SD + SiNPs-treated groups also differed significantly from the NC group **(**Figs. 2A-B**)**. The core body temperature of the NC group followed a normal circadian pattern, increasing with locomotor activity during the dark cycle **(**Fig. 2C**)**. In contrast, the SiNPs-treated and SD + SiNPs-treated group showed an abnormal pattern, their core body temperature was lower than the NC group in both the light and dark cycles **(**Figs. 2C-D**)**.

To assess the effect of SD on the general behaviors of the mice, we collected data on pellet intake, water intake, and body weight over a two-week period. We observed no significant statistical differences in water intake and pellet intake among the three groups (Fig. 2E-F). Pellet and water intake were slightly lower in the SD + SiNPs-treated group compared to the NC group, but the difference was not statistically significant. The SiNPs-treated group exhibited the lowest intake among the three groups, yet these reductions were also not statistically significant (Fig. 2E-F). Despite the lack of significant differences in water intake, there was a clear trend in both pellet and water consumption, as well as a significant difference in body weight, with the SD + SiNPs-treated group weighing less than the other two groups **(**Fig. 2G**)**.

Collectively, these results demonstrate that SD significantly exacerbates SiNPs-induced disturbances in circadian behavior and physiology, as evidenced by alterations in locomotor activity, core temperature regulation, and body weight.

### 3.2 Structural alterations in the ELGs triggered by sleep deprivation in SiNPs-treated mice

Our previous studies have shown that the size and weight of ELGs exhibit circadian oscillations, which can be modulated by both intrinsic (e.g., circadian disruption) [33] and extrinsic factors (e.g., type 1 diabetes) [48]. Building upon these findings, we investigated whether exposure to SiNPs and SD, individually or in combination, could alter the physiological characteristics of ELGs, particularly their structure and function.

**Fig. 3:**
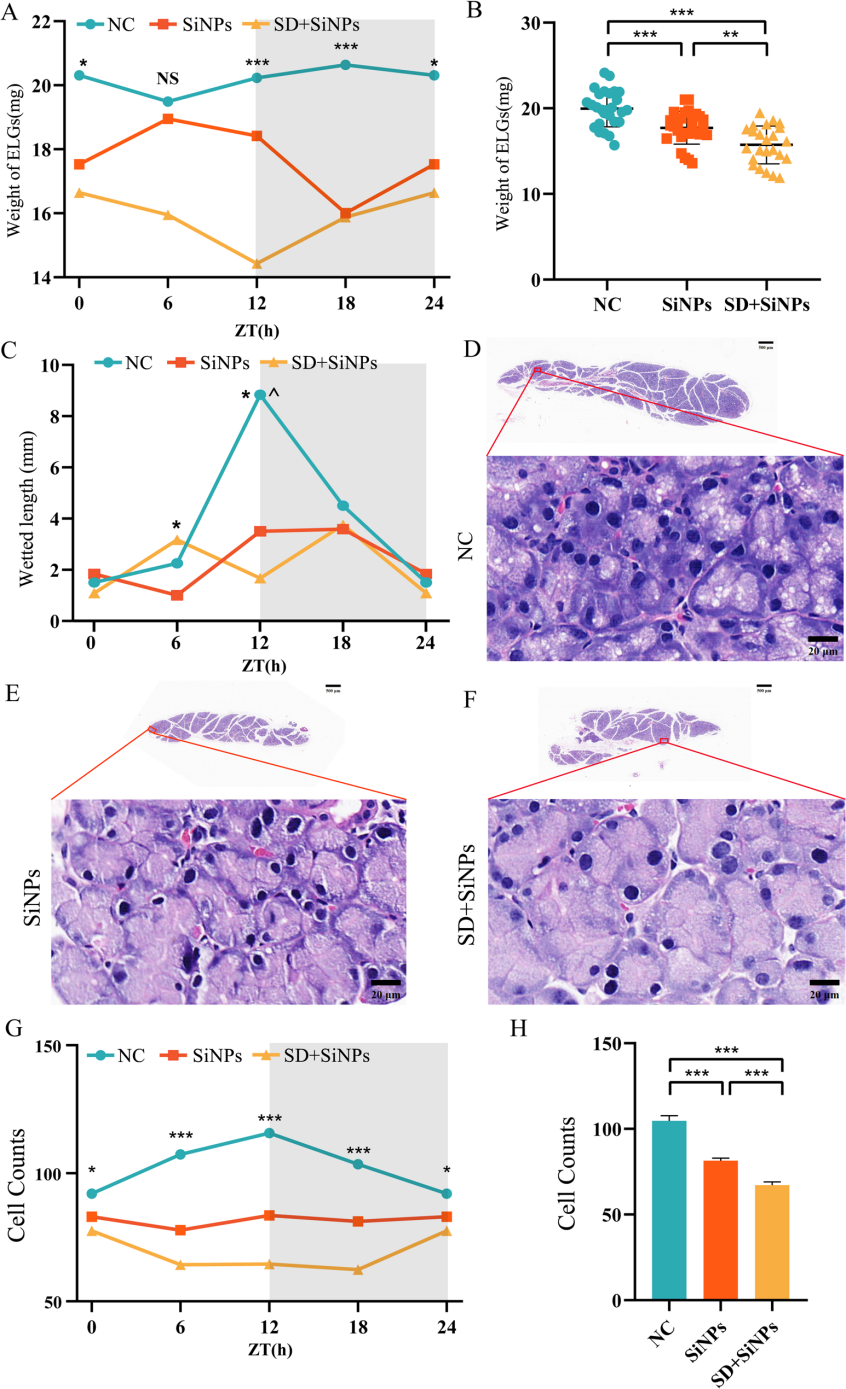
Impact of SiNPs and SD + SiNPs treatment on ELG weight, tear secretion, and structural integrity. **(A)** Diurnal changes of ELG weight in the NC, SiNPs-treated, and SD + SiNPs-treated groups. ^*^P < 0.05, ^***^P < 0.001. NS: not significant. (**B**) ELG weight measurements in the NC, SiNPs-treated, and SD + SiNPs-treated groups. ^**^P < 0.01, ^***^P < 0.001. **(C)** Tear secretion was assessed using the phenol thread test at ZT 0, 6, 12, and 18 over a 24-hour cycle. Statistical significance is denoted as ^*^P < 0.05 for comparisons between SiNPs-treated and SD + SiNPs-treated groups and ^*^*^*P* < 0.05 for comparisons between the NC and SD + SiNPs-treated groups. **(D-F)** Representative gross sections of ELGs from the NC (*D*), SiNPs-treated (*E*), and SD + SiNPs-treated (*F*) groups (scale bar: 500 μm, upper panels). Magnified views of the boxed regions show the acinar cell morphology (scale bar: 20 μm, lower panels). **(G)** Diurnal changes of ELG cell counts in the NC, SiNPs-treated, and SD + SiNPs-treated groups. ^*^P < 0.05, ^***^P < 0.001. **(H)** Quantitative analysis of acinar cell counts in the NC, SiNPs-treated, and SD + SiNPs-treated groups. Statistical analysis was performed using Brown-Forsythe ANOVA. Multiple comparisons were conducted using the Games-Howell post hoc test. ^***^P < 0.001

ELG weights were measured at four ZT points (ZT0, ZT6, ZT12, and ZT18) across all experimental groups. In the NC group, ELG weight displayed robust diurnal variation, peaking at ZT18. This rhythmicity was abolished in both the SiNPs-treated and SD + SiNPs-treated groups. Notably, SD further exacerbated the disruption caused by SiNPs **(**Fig. 3A**)**. Overall, ELG weights were significantly lower in both treatment groups compared to NC, indicating compromised lacrimal gland homeostasis **(**Fig. 3B**)**.

Tear secretion, was assessed using phenol red thread tests, also followed a circadian rhythm in the NC group, with a peak at ZT18. This rhythmic pattern was absent in the SiNPs-treated and SD + SiNPs-treated groups. Importantly, no statistically significant differences in tear volume were observed between the two treatment groups, suggesting that SiNPs-induced dysfunction had already reached a plateau, with SD **(**Fig. 3C**)**.

To assess structural changes in the lacrimal gland, HE staining was performed for histopathological examination **(**Figs. 3D-F**)**. Figure 3G shows daily oscillations in the NC group, which is disrupted in both the SiNPs-treated and SD + SiNPs-treated groups. Quantitative analysis in Fig. 3H revealed significant differences in the number of cell nuclei among the three groups. The number of nuclei was significantly reduced in the SiNPs-treated group compared to the NC group, and further decreased in the SD + SiNPs-treated group relative to both the NC group and the SiNPs-treated group. These findings suggest that both SiNPs exposure and the combined intervention with SD induce structural abnormalities and functional impairment in ELGs, with more severe effects observed in the SD + SiNPs-treated group.

Collectively, these findings demonstrate that SD intensifies SiNPs-evoked disruptions in locomotor activity, core temperature regulation, and body weight.

### 3.3 Alterations in global gene expression of ELGs promoted by sleep deprivation in SiNPs-treated mice

To investigate the oscillations in the transcriptome of murine ELGs, we collected ELGs at three-hour intervals throughout a 24-hour period from the NC, SiNPs-treated, and SD + SiNPs-treated groups of C57BL/6J mice. RNA-seq was performed on the BGISEQ-500 platform, and transcriptome analysis was conducted using the JTK_CYCLE algorithm with a 24-hour oscillation period and a significance threshold of *P* < 0.05.

**Fig. 4:**
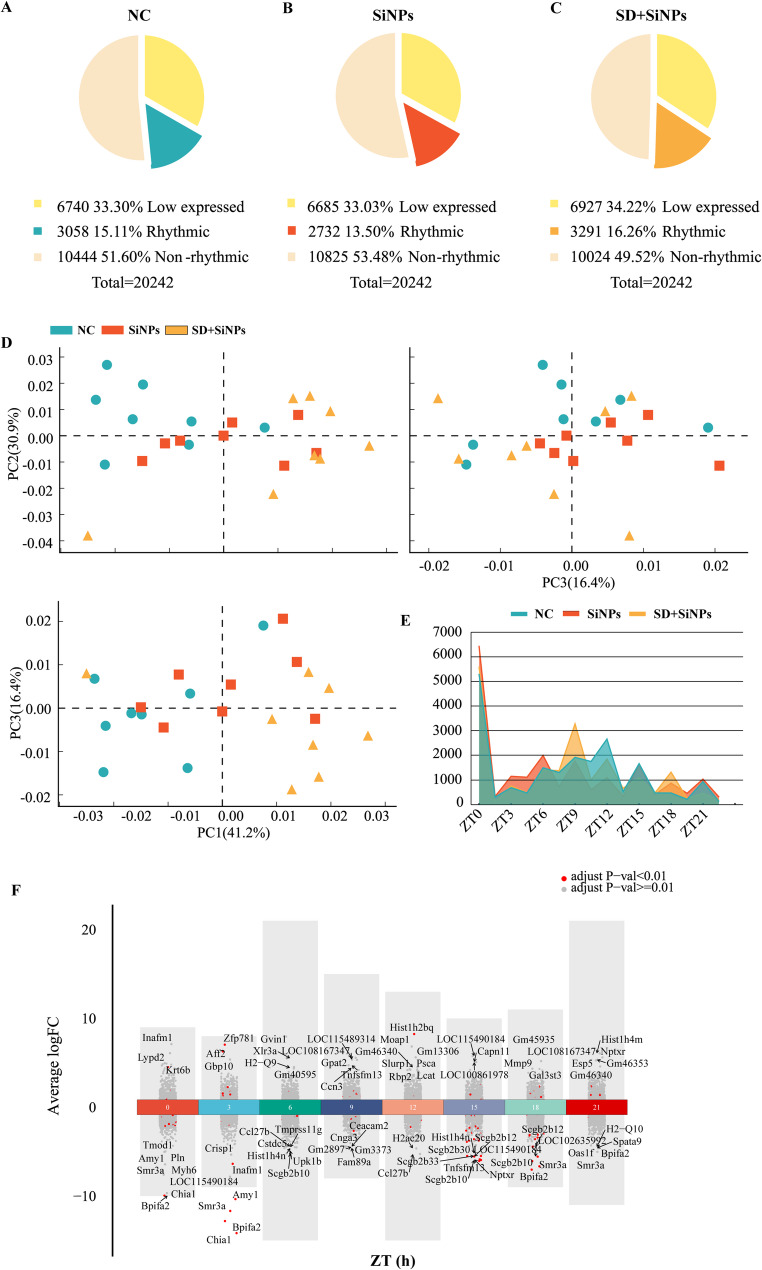
Comparative transcriptomic analysis of mouse ELGs in response to SiNPs and SD + SiNPs treatments. **(A–C)** The pie charts illustrate the transcriptomic composition of ELGs in the NC, SiNPs-treated, and SD + SiNPs-treated groups, categorized into low-expressed, rhythmic, and non-rhythmic genes. **(D)** The PCA scatterplot depicts the overall gene expression profiles of ELGs in the NC (blue), SiNPs-treated (red), and SD + SiNPs-treated (yellow) groups. Each dot represents an individual animal sampled at three-hour intervals over a 24-hour cycle (*N* = 3 per time point). The shaded regions indicate the distribution of each group. **(E)** The line graph illustrates the temporal distribution of peak gene expression across different ZT points in the NC, SiNPs-treated, and SD + SiNPs-treated groups. Differences in peak expression times highlight the impact of treatments on rhythmic gene regulation. **(F)** The volcano plot visualizes DEGs in ELGs between NC and SiNPs-treated mice. The x-axis represents ZT points, while the y-axis indicates fold change (FC). Red and gray dots denote genes with adjusted *P*-values < 0.01 and ≥ 0.01, respectively. Each group consisted of 24 mice

A total of 20,242 genes were identified through RNA-seq analysis. Genes were categorized into rhythmic (FPKM ≥ 0.1, adjusted *P* < 0.05), non-rhythmic (FPKM ≥ 0.1, adjusted *P* ≥ 0.05), and low-expressed (FPKM < 0.1) based on the obtained data. Among the 20,242 transcripts, rhythmic genes constituted 15.10%, 13.50%, and 16.26% of the NC, SiNPs-treated, and SD + SiNPs-treated groups, respectively. Low-expressed genes accounted for 33.30%, 33.02%, and 34.22% in the respective groups. Non-rhythmic genes were present in 51.60%, 53.48%, and 49.52% of the NC, SiNPs-treated, and SD + SiNPs-treated groups (Figs. 4A-C).

Compared to the NC group, the SiNPs-treated group experienced a decrease of 0.27% in low-expressed genes and 1.61% in rhythmic genes, whereas the SD + SiNPs-treated group showed an increase of 0.92% in low-expressed genes and 1.16% in rhythmic genes. The proportion of non-rhythmic genes increased by 1.88% in the SiNPs-treated group and decreased by 2.48% in the SD + SiNPs-treated group relative to NC (Figs. 4A-C).

Principal component analysis (PCA) was employed to discern distinct groupings among the treatments. The first three principal components accounted for 41.2%, 30.9%, and 16.4% of the variance, respectively (Fig. 4D). The PCA biplots demonstrated that the NC, SiNPs-treated, and SD + SiNPs-treated groups formed three distinct clusters, indicating that SiNPs and SD + SiNPs treatments have a significant impact on differential gene expression.

The distribution of gene expression peaks over a 24-hour period revealed the effects of SiNPs and SD + SiNPs treatments (Fig. 4E). The NC group exhibited peak gene expression at ZT12 and ZT15. In contrast, the SiNPs-treated group displayed peaks at ZT0, ZT3, ZT6, and ZT21. The SD + SiNPs-treated group showed peak expression at ZT9 and ZT18.

Volcano plot effectively highlights the top10 significantly differentially expressed genes (DEGs) between the NC group and the SiNPs-treated group (Fig. 4F and Table S1), providing insights into the effect of SiNPs treatment on ELGs. Volcano plots of significant DEGs between the NC group and the SD + SiNPs-treated, and between the SiNPs-treated and the SD + SiNPs-treated, are presented in Figure S2 and Tables S2-3.

Overall, these findings demonstrate SiNPs exposure—accentuated by SD—substantially alters the ELG transcriptome, differentially affecting rhythmic and non-rhythmic genes and highlighting the circadian sensitivity of the lacrimal gland to environmental and behavioral stressors.

### 3.4 Disruption of circadian rhythmicity of ELGs promoted by sleep deprivation in SiNPs-treated mice

**Fig. 5:**
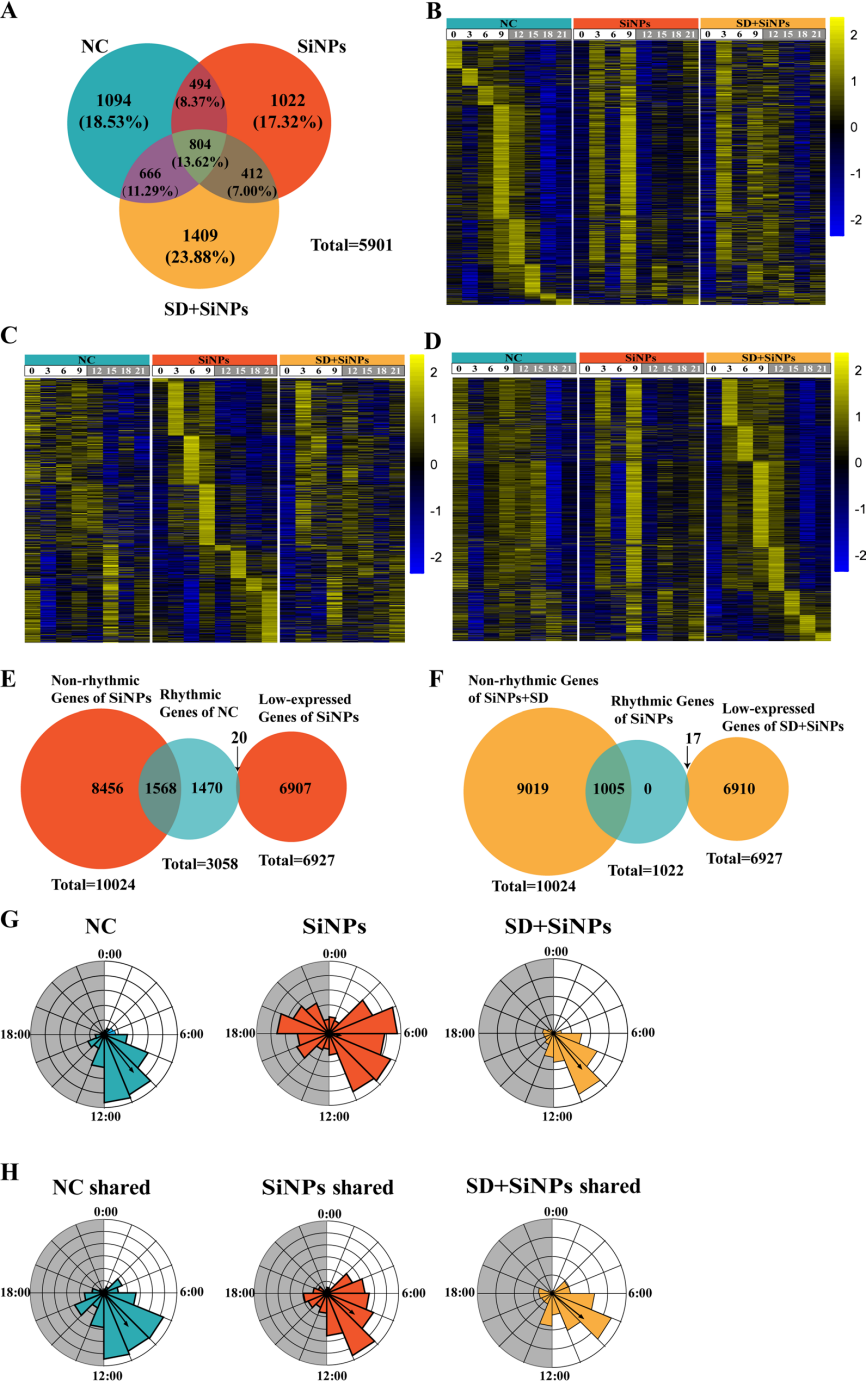
Effects of SiNPs and SD + SiNPs treatment on the rhythmic transcriptome of murine ELGs. **(A)** The venn diagram illustrates the overlap and divergence of rhythmic transcripts among the NC, SiNPs-treated, and SD + SiNPs-treated groups. **(B)** The heatmaps display the expression profiles of 1,094 rhythmic transcripts unique to the NC group at different ZT points. The left panel represents the NC group, while the middle and right panels correspond to the SiNPs-treated and SD + SiNPs-treated groups, respectively. Gene expression levels are normalized within a ± 2 range, as indicated by the color scale. **(C)** The heatmaps show the expression levels of 1,022 rhythmic transcripts exclusive to the SiNPs-treated group across various ZT points. The panel arrangement is consistent with (*B*), with the NC group on the left, the SiNPs-treated group in the middle, and the SD + SiNPs-treated group on the right. **(D)** The heatmaps illustrates the expression patterns of 1,409 rhythmic transcripts unique to the SD + SiNPs-treated group over multiple ZT points, following the same panel arrangement as in (*B*) and (*C*). **(E)** The venn diagram depicts the transition of rhythmic genes in the NC group to either non-rhythmic (blue) or low-expressed (red) genes in the SiNPs-treated group. **(F)** The venn diagram illustrates the conversion of rhythmic genes in the SiNPs-treated group into non-rhythmic (red) or low-expressed (yellow) genes in the SD + SiNPs-treated group. **(G-H)** The wind rose diagrams represent the mean vector and length of rhythmic genes unique to each group *(G)* and those shared across all three groups *(H)*. The NC, SiNPs-treated, and SD + SiNPs-treated groups are positioned on the right, middle, and left, respectively

To explore the impact of SiNPs treatment and SD + SiNPs treatment on the circadian rhythmicity of ELGs, we employed Venn diagrams, heatmaps, pie charts, and rose diagrams to analyze the rhythmic genes among the three groups. Venn diagrams were utilized to identify unique and shared rhythmic genes across the NC, SiNPs-treated, and SD + SiNPs-treated groups. Among the 5,901 non-redundant gene transcripts, the proportion of unique rhythmic genes in each group was 18.53% for the NC, 17.32% for the SiNPs-treated, and 23.88% for the SD + SiNPs-treated groups (Fig. 5A and Table S4).

Heatmaps were constructed to illustrate the expression levels of 1,096 unique genes in the NC group (Figs. 5B), 1,022 in the SiNPs-treated group (Figs. 5C), and 1,409 in the SD + SiNPs-treated group (Figs. 5D) across eight ZT points. The heatmaps revealed significant alterations in gene expression patterns between the SiNPs-treated group and the NC group, with further pronounced changes observed in the SD + SiNPs-treated group.

Pie charts were used to depict the transformation of rhythmic genes post-treatment (Figs. 5E-F). Of the rhythmic genes unique to NC group, 1,470 maintained their rhythmic expression after SiNPs treatment, while 1,568 transitioned to non-rhythmic status (Fig. 5E). In the SiNPs-treated group, none of the unique rhythmic genes remained unchanged after SD + SiNPs treatment; 1,005 became non-rhythmic, and 17 became low-expressed (Fig. 5F).

The rose diagrams, generated using Oriana software, visually demonstrated the differences in the periodicity and phase distribution of unique and shared rhythmic genes across groups. The NC group exhibited a mean vector (*µ*) of 9:30 and a mean vector length (r) of 0.635, the SiNPs-treated group showed *µ* = 6:40 and *r* = 0.163, and the SD + SiNPs-treated group presented µ = 9:26 and *r* = 0.596 (Fig. 5G). Figure 5H illustrates the changes in the average vector and its length for shared rhythmic genes under different treatments: NC group (*µ* = 9:37, *r* = 0.55), SiNPs-treated group (*µ* = 8:29, *r* = 0.463), and SD + SiNPs-treated group (*µ* = 8:34, *r* = 0.532).

The composite pie charts revealed phase shifts among shared genes across groups. After SiNPs treatment, 33.83% of shared genes in the NC group maintained their phase, while 66.17% experienced phase shifts, with 68.98% advancing and 31.02% delaying. Following SD + SiNPs treatment, 34.45% of shared genes remained phase-stable, 65.55% underwent phase changes, with 43.83% delaying and 56.17% advancing. In the SD + SiNPs treatment group, 82.71% of shared genes exhibited phase alterations, 66.32% advanced, and 33.68% delayed, with only 17.29% remaining unchanged (Fig. S1).

The findings indicate that both SiNPs treatment and the combination of SD + SiNPs treatment significantly disrupt the circadian rhythmicity of ELGs, leading to substantial alterations in the expression and phase distribution of rhythmic genes, which underscores the profound impact of these treatments on the molecular clock of lacrimal gland function.

### 3.5 Reshaped KEGG and phase-set enriched pathways triggered by sleep deprivation in SiNPs-treated mice

**Fig. 6:**
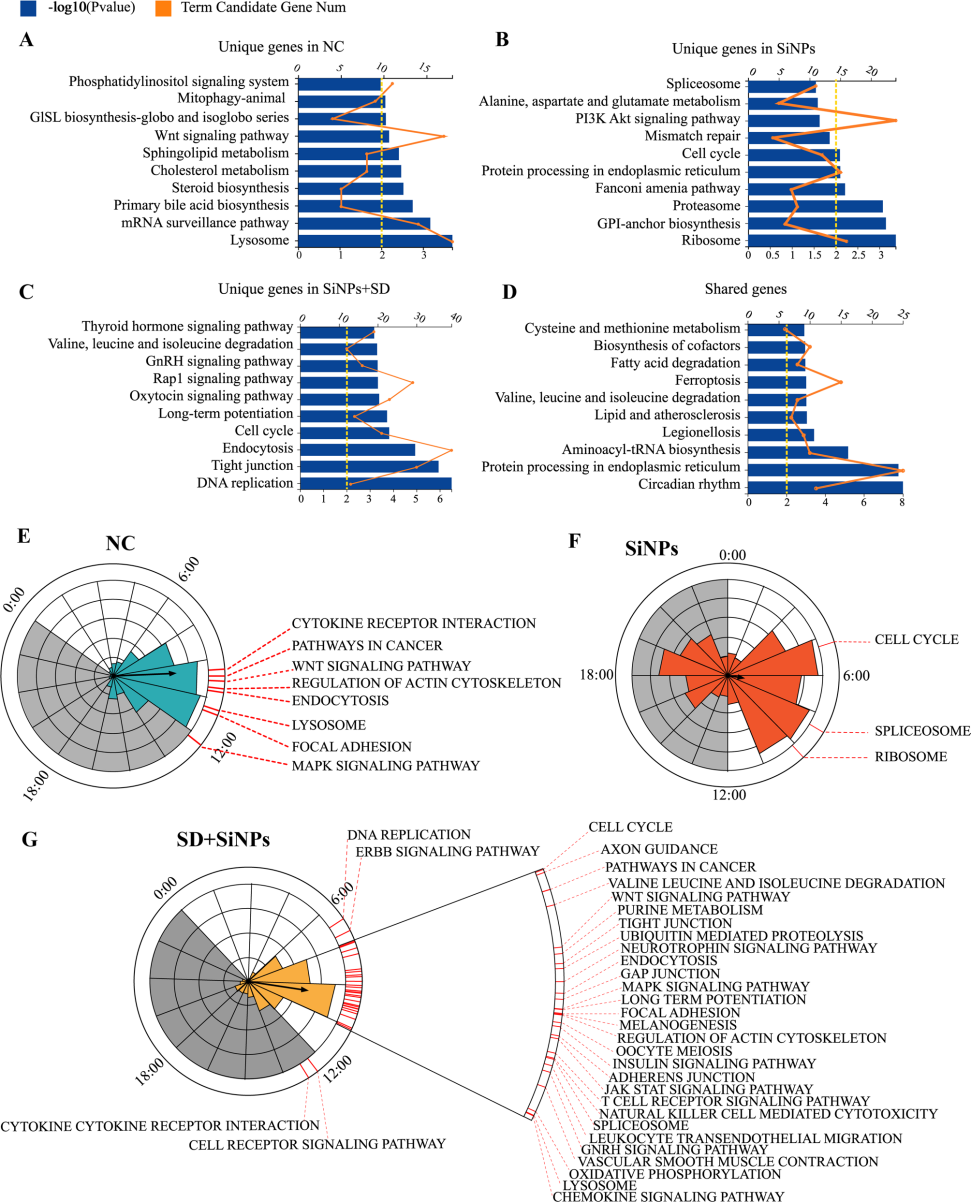
Impact of SiNPs and SD + SiNPs treatment on KEGG and phase-clustered pathways in mouse ELGs. **(A–D)** Gene annotation of KEGG pathways significantly enriched in rhythmic genes unique to the NC group (*A*), SiNPs-treated group (*B*), SD + SiNPs-treated group (*C*), and those shared among all three groups (*D*), with *P* < 0.01. The top 10 enriched pathways are presented. The upper horizontal axis, aligned with the orange line graph, represents the number of term candidate genes, while the lower horizontal axis, aligned with the blue histogram, represents the -log10 (*P*-value), indicating the statistical significance of enrichment. **(E–G)** Summary of significantly phase-clustered pathways (*P* < 0.05) unique to the NC group *(E)*, SiNPs-treated group *(F)*, and SD + SiNPs-treated group *(G)*. The inner circle and column length represent the phase distribution of rhythmic genes specific to each group. The outer red line marks KEGG pathways (*P* < 0.05) associated with rhythmic genes unique to each group, indicating enrichment at distinct ZT points, as determined by the phase distribution in the inner circle. Gray shading indicates dark cycles

To evaluate the functional implications of circadian gene expression changes, KEGG pathway enrichment analysis was performed on rhythmic genes unique to the NC, SiNPs-treated, and SD + SiNPs-treated groups. The genes exclusive to the NC group were significantly enriched in nine pathways (Fig. 6A and Table S5, *P* < 0.05), with a focus on cellular processes and metabolic pathways. However, the rhythmic genes of SiNPs-treated group enriched in different KEGG pathways from the NC group (Fig. 6B and Table S6, *P* < 0.05), so did the rhythmic genes of SD + SiNPs-treated group (Fig. 6C and Table S7, *Q* < 0.05). Notably, the cell cycle pathway was the only pathway shared between the SiNPs-treated and SD + SiNPs-treated groups.

A total of 18 pathways were enriched among genes common to all groups (Fig. 6D and Table S8, *Q *< 0.05). The top 10 pathways identified for each group were categorized into five groups: Cellular Processes, Genetic Information Processing, Metabolism, Organismal Systems, and Environmental Information Processing. The SiNPs-treated group’s pathways fell within Cellular Processes, Genetic Information Processing, and Metabolism (Table S5). After the SD + SiNPs treatment, the enriched pathways expanded to include all five categories (Table S6). Although the SD + SiNPs-treated group’s unique pathways matched the control group’s categories, the SD + SiNPs treatment did not fully counteract the effects of SiNPs treatment (Table S7). According to KEGG pathway level 2, the biological process categories for the SD + SiNPs-treated group diverged from those of the NC and SiNPs-treated groups, suggesting that SD + SiNPs treatment may amplify the effects of SiNPs treatment.

PSEA was employed to investigate the temporal expression patterns of these pathways. The NC group’s significantly enriched pathways were predominantly expressed between ZT9 and ZT12 (Fig. 6E and Table S9, Kuiper *Q* < 0.05). In contrast, the SiNPs-treated group had three pathways peaking near ZT4, ZT5, and ZT9 (Fig. 6F and Table S10, Kuiper *Q* < 0.05). The SD + SiNPs-treated group displayed enriched pathway expression primarily between ZT6 and ZT12, with two pathways, B cell receptor signaling and cytokine receptor interaction, exhibiting expression during the dark phase (Fig. 6G and Table S11, Kuiper *Q* < 0.05).

Overall, these findings demonstrate that SiNPs disturb the phase distribution of multiple ELG pathways, and SD broadens and deepens these shifts, showing that combined environmental and behavioral stressors can seriously compromise circadian and functional homeostasis in the lacrimal gland.

### 3.6 Alterations in cluster-dependent transcriptomic map and KEGG pathways triggered by sleep deprivation in SiNPs-treated mice

**Fig. 7:**
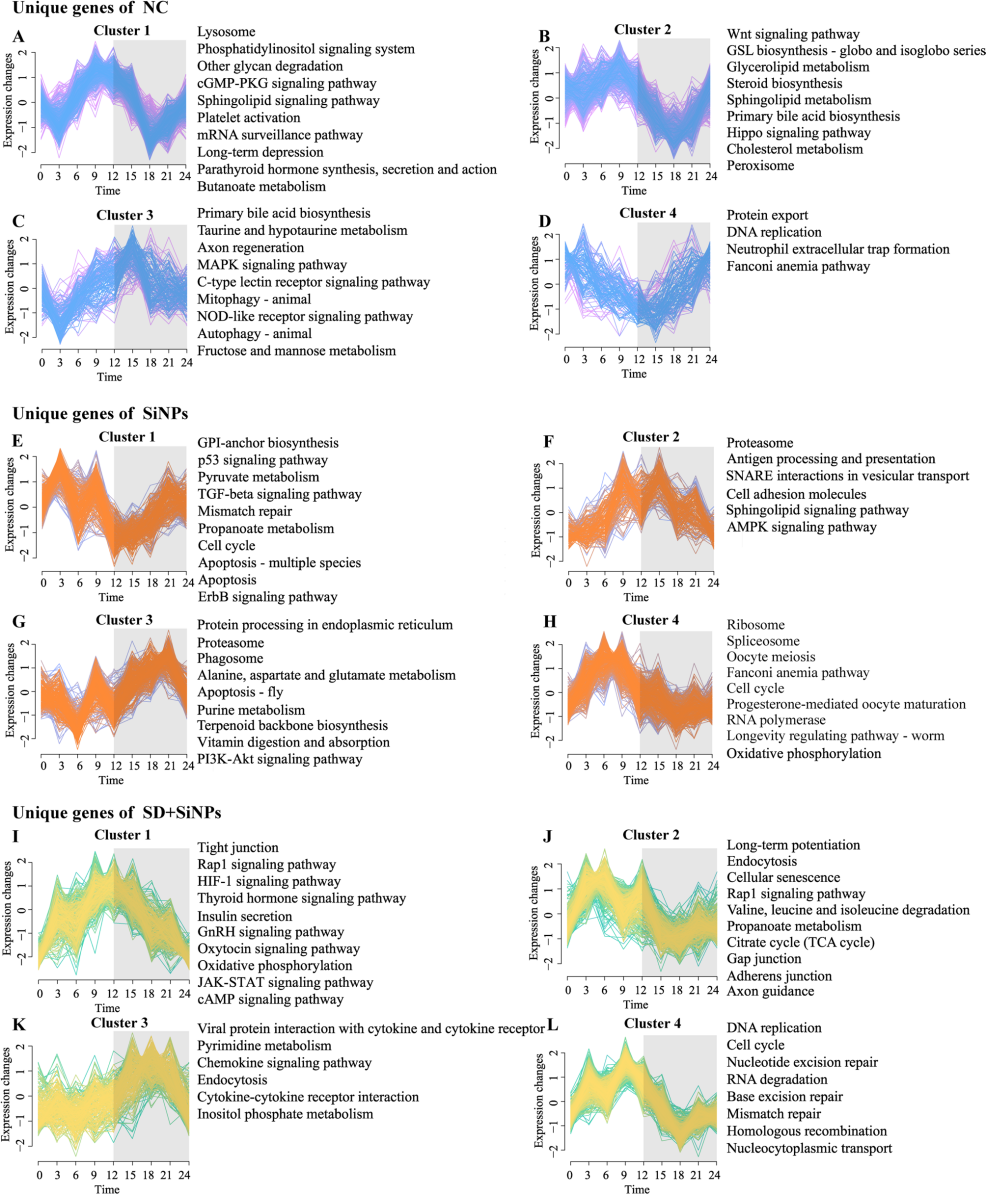
Impact of SiNPs and SD + SiNPs treatment on the circadian gene clustering profile and KEGG pathways in murine ELGs. **(A–L)** Temporal gene expression Z-scores for four distinct enriched clusters unique to the NC group *(A–D)*, SiNPs-treated group *(E–H)*, and SD + SiNPs-treated group *(I–L)*. Blue, orange, and yellow lines represent genes with low membership values, while pink, purple, and green lines indicate genes with high membership values. Gray shading denotes dark cycles. The corresponding right-side panels display the top 10 KEGG pathways (*P* < 0.05) enriched for circadian genes unique to each group and cluster

To investigate the temporal trends in rhythmic gene expression, we applied the Mfuzz soft clustering package for analysis. Our findings revealed that genes from the NC group (Figs. 7A-D), the SiNPs-treated group (Figs. 7E-H), the SD + SiNPs-treated group (Figs. 7I-L) were each categorized into four distinct clusters, each exhibiting unique expression profiles and enriched pathways.

In Cluster 1, significant disparities were observed among the NC, SiNPs-treated, and SD + SiNPs-treated groups, with gene counts of 439, 242, and 289, respectively. The NC group exhibited a notably higher number of clustered genes. The genes in the NC group peaked at ZT9 and troughed at ZT3 and ZT18 **(**Fig. 7A, ***left*)**. In contrast, the SiNPs-treated group peaked at ZT3 and troughed at ZT12 **(**Fig. 7E, ***left***), whereas the SD + SiNPs-treated group peaked at ZT12 **(**Fig. 7I, ***left***). These distinct global patterns underscore the divergent regulatory mechanisms at play.

Cluster 2 showed 367, 157, and 503 genes in the NC, SiNPs-treated, and SD + SiNPs-treated groups, respectively. The NC and SD + SiNPs-treated groups shared similar expression patterns, peaking at ZT9 and troughing at ZT18 **(**Figs. 7B and F, ***left***). However, the SiNPs-treated group deviated from this trend, peaking at ZT12 and troughing at ZT3 **(**Fig. 7J, ***left***).

The gene expression patterns in Cluster 3 were largely concordant across the groups, with gene counts of 165, 269, and 231, respectively, albeit with variations in the timing of peaks and troughs **(**Figs. 7C, G and K, ***left*)**. In Cluster 4, the SiNPs-treated and SD + SiNPs-treated groups exhibited similar gene counts (358 and 392, respectively) **(**Figs. 7H and L, ***left*)**, in contrast to the NC group, which had a significantly lower count of 127 **(**Fig. 7D, ***left*)**. The expression patterns of enriched genes in the SiNPs-treated and SD + SiNPs-treated groups were broadly similar but diverged from those of the NC group.

Furthermore, the KEGG pathways enriched in genes belonging to each cluster varied across groups, indicating a reprogramming of the transcriptomic landscape in response to SiNPs treatment and SD **(**Figs. 7A-L, ***right*)**. The KEGG pathways associated with the NC, SiNPs-treated, and SD + SiNPs-treated groups, along with their respective clusters, are shown in Tables S12-14, respectively. This finding suggests a complex interplay between these factors in modulating the biological pathways of ELGs.

In summary, soft clustering and KEGG pathway analysis revealed that SD, particularly when combined with SiNPs exposure, reprograms the temporal architecture of the ELG transcriptome. These alterations reflect a complex interaction between environmental and behavioral stressors and have profound implications for the regulation of lacrimal gland function and circadian homeostasis.

### 3.7 Alterations in core clock genes of ELGs promoted by sleep deprivation in SiNPs-treated mice

**Fig. 8:**
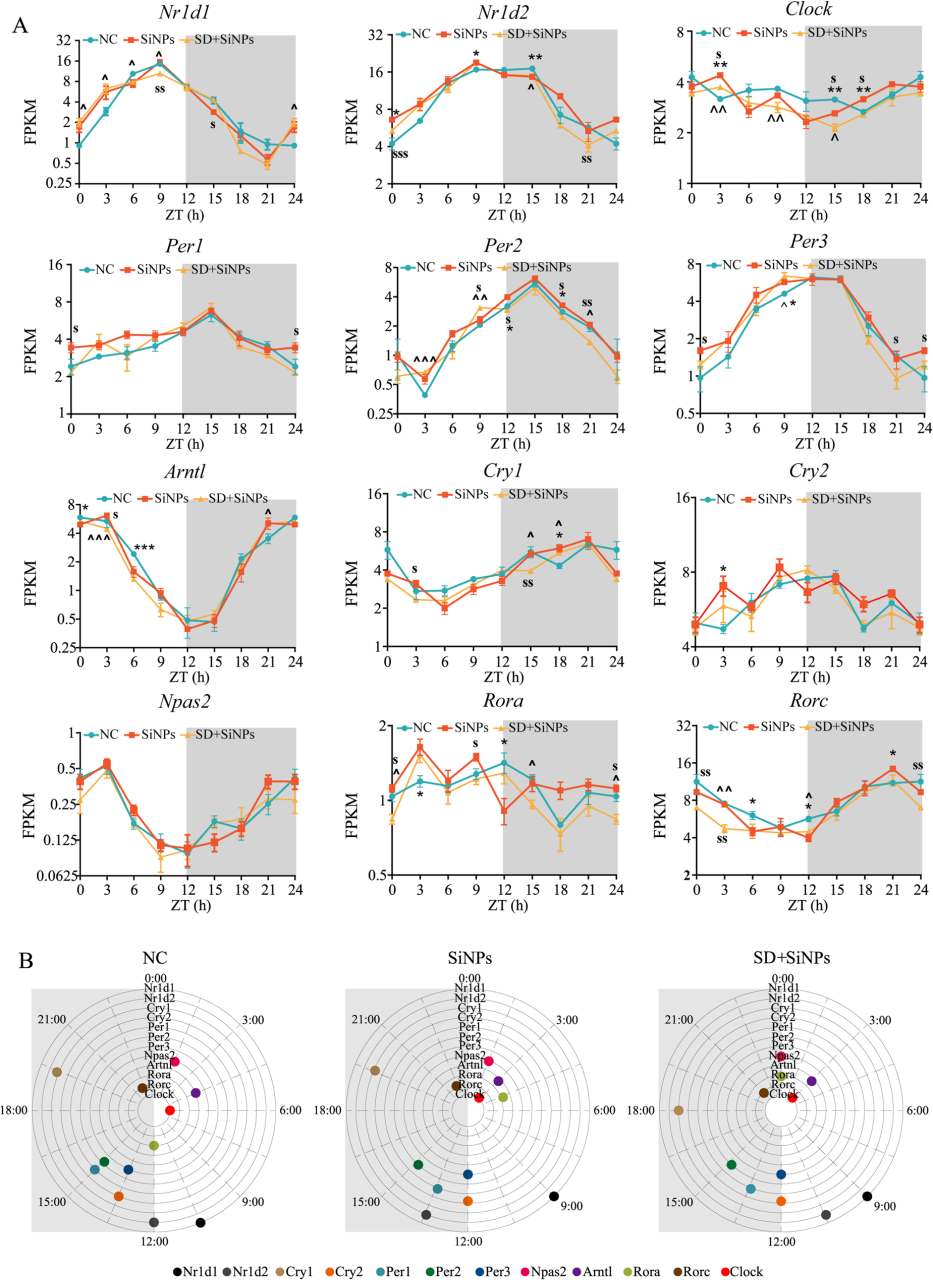
Impact of SiNPs and SD + SiNPs treatment on circadian transcription in murine ELGs. **(A)** The 24-hour expression profiles of 12 core clock genes, including *Nr1d1* (*REV-ERBα*), *Nr1d2* (*REV-ERBβ*), *Clock*,* Per1*,* Per2*,* Per3*,* Arntl* (*Bmal1*), *Cry1*,* Cry2*,* Npas2*,* Rora*, and *Rorc*, are presented. The x-axis represents the sampling time points, while the y-axis indicates gene expression levels at specific ZT points. The green, orange, and yellow lines correspond to the NC, SiNPs-treated, and SD + SiNPs-treated groups, respectively. Gray shading denotes the dark phase of the LD cycle. Three animals per group were sampled every three hours. At each time point, independent samples *t*-tests were applied to assess differences between the NC and SiNPs-treated groups, the NC and SD + SiNPs-treated groups, and the SiNPs-treated and SD + SiNPs-treated groups. Statistical significance is indicated as follows: ^*^P < 0.05, ^**^P < 0.01, ^***^P < 0.001 for comparisons between the NC and SiNPs-treated groups; ^*^*^*P* < 0.05, ^*^^*^*P* < 0.01, ^*^^^*^*P* < 0.001 for comparisons between the NC and SD + SiNPs-treated groups; and ^*s*^*P* < 0.05, ^*ss*^*P* < 0.01, ^*sss*^*P* < 0.001 for comparisons between the SiNPs-treated and SD + SiNPs-treated groups. **(B)** The distribution of peak phases for core clock genes is shown for the NC, SiNPs-treated, and SD + SiNPs-treated groups. Gray shading represents the dark phase of the circadian cycle

To investigate the effects of SiNPs and SD on the molecular circadian machinery of the lacrimal glands, we analyzed the temporal expression profiles of 12 core clock genes (*Nr1d1*, *Nr1d2*, *Clock*, *Per1*,* Per2*, *Per3*, *Arntl*, *Cry1*, *Cry2*, *Npas2*, *Rora*, and *Rorc*) across the NC, SiNPs-treated, and SD + SiNPs-treated groups**(**Fig. 8A**)**. A two-way repeated measures ANOVA was performed to evaluate the effects of group, time point, and their interaction on gene expression levels. The results are summarized in Table S15. A significant main effect of time was observed for *Per3*, *Cry2*, and *Npas2*, while no significant effect of group or group × time interaction was found. These results indicate that gene expression varied over time, consistent with circadian regulation, but was not significantly affected by SiNPs or SD treatments. For *Nr1d1*, *Arntl*, *Per1*, *Cry1*, and *Rora*, significant main effects of time and group × time interaction were detected, but not for group alone, indicating that temporal expression dynamics differed between treatments despite similar overall expression levels. Although significant main effects of group, time, and group × time interaction were observed for *Nr1d2*, *Clock*,* Per2*, and *Rorc*, post hoc comparisons between groups did not reveal statistically significant differences. These findings suggest that treatment influenced the temporal expression dynamics of these genes, but differences between groups were relatively modest and did not reflect persistent changes in average expression levels. A separate two-way ANOVA confirmed that treatment, time point, and their interaction significantly influenced gene expression levels. Independent-sample *t*-tests at individual time points showed that, although not all comparisons reached significance, both SiNPs and SD + SiNPs treatments independently or synergistically affected gene expression at several time points. Furthermore, the SD + SiNPs-treated group exhibited distinct expression patterns compared to the SiNPs-treated group at several time points.

Figure 8B illustrates the expression phases of the core clock genes across the three groups. Following SiNPs treatment and SD + SiNPs treatment, the expression phases of 11 out of 12 core clock genes, excluding *Per2*, were altered relative to the NC group. Specifically, the phases of *Nr1d1*,* Nr1d2*,* Clock*,* Per1*,* Per3*, and *Cry2* were affected by SiNPs treatment and remained consistent with those of the SiNPs-treated group following SD + SiNPs treatment. The phases of *Rorc*,* Arntl*,* Cry1*, and *Npas2* remained unchanged after SiNPs treatment but shifted following SD + SiNPs treatment. The phase of *Rora* was influenced by both treatments. Specifically, in the NC group, four clock components are present in the light cycle, exhibiting four peak phases: 01:30 (*Npas2*), 04:30 (*Artnl*), 06:00 (*Clock*), and 10:30 (*Nr1d1*). Six clock components are found in the dark cycle, with peak phases at 13:30 (*Cry2* and *Per3*), 15:00 (*Per1* and *Per2*), 19:30 (*Cry1*), and 22:30 (*Rorc*). Additionally, two peak phases at 12:00 (*Rora* and *Nr1d2*). Furthermore, in the SiNPs-treated group, five clock components are distributed in the light cycle, with four peak phases: 01:30 (*Npsa2*), 03:00 (*Clock* and *Artnl*), 04:30 (*Rora*), and 09:00 (*Nr1d1*). In the dark cycle, five clock components are present, showing four peak phases at 13:30 (*Per1* and *Nr1d2*), 15:00 (*Per2*), 19:30 (*Cry1*), and 22:30 (*Rorc*). At the transition between the light and dark cycles, two clock components are observed, peaking at 12:00 (*Per3* and *Cry2*). Moreover, in the SD + SiNPs-treated group, four clock components are found in the light cycle, with three peak phases at 03:00 (*Clock* and *Artnl*), 09:00 (*Nr1d1*), and 10:30 (*Nr1d2*). In the dark cycle, four clock components show peak phases at 13:30 (*Per1*), 15:00 (*Per2*), 18:00 (*Cry1*), and 22:30 (*Rorc*). At the junction of the light and dark cycles, four clock components peak at 00:00 (*Npas2* and *Rora*) and 12:00 (*Cry2* and *Per3*).

Collectively, these findings suggest that while SiNPs and SD + SiNPs treatments do not drastically alter the average expression levels of core circadian genes, they significantly affect their phase relationships and temporal dynamics. This reprogramming of clock gene oscillations highlights a nuanced yet critical mode of circadian disruption within the ELGs under environmental and behavioral stress.

### 3.8 Immune cell infiltration triggered by sleep deprivation in SiNPs-treated mice

**Fig. 9:**
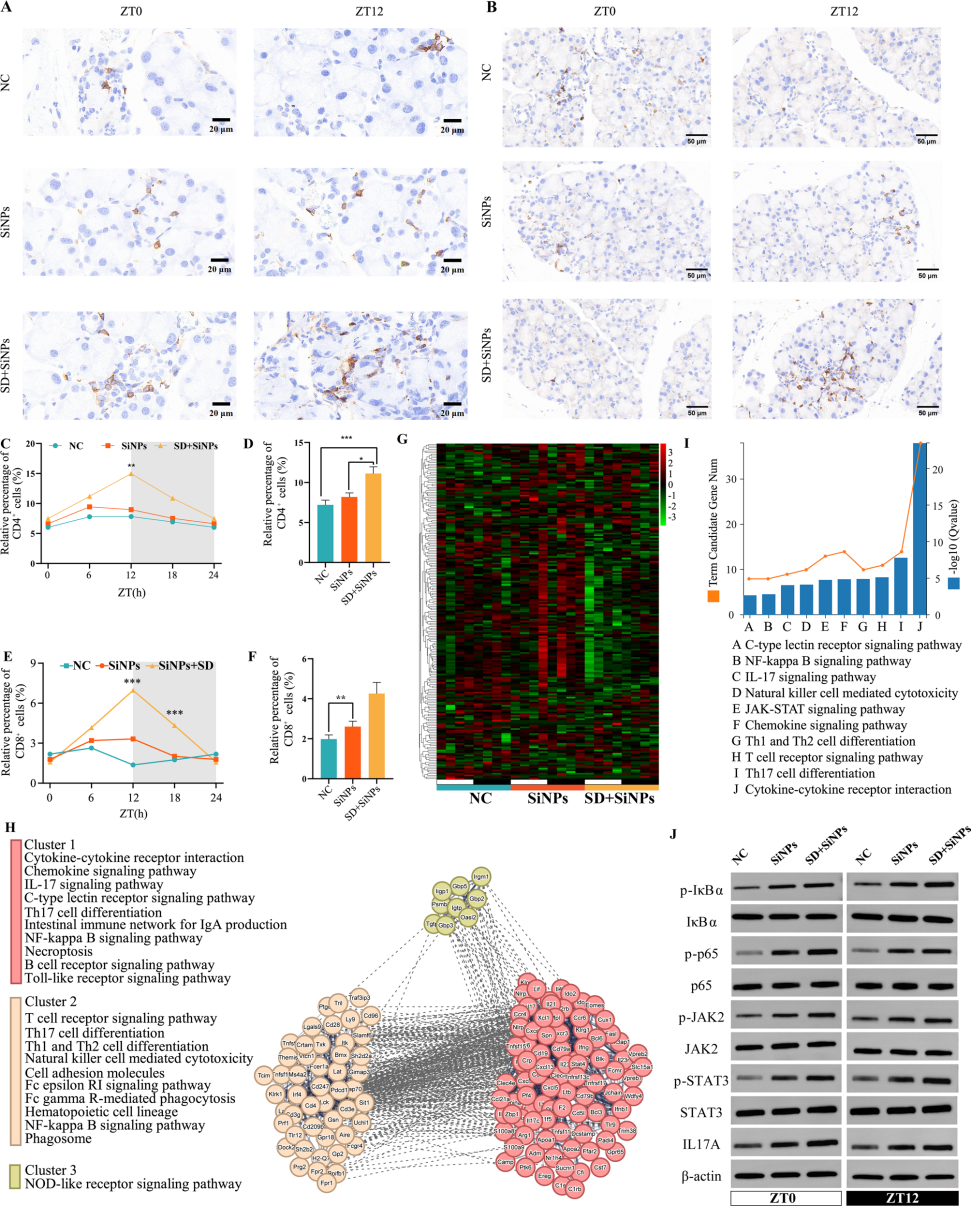
Impact of SiNPs and SD + SiNPs treatment on immune cells and genes in murine ELGs. **(A)** Representative immunohistochemical images of CD4^+^ T cells in murine ELGs at ZT0 and ZT12, from NC, SiNPs-treated and SiNPs + SD-treated groups. *Scale bar*: 20 μm. **(B)** Representative immunohistochemical images of CD8^+^ T cells in murine ELGs at ZT0 and ZT12, from the NC, SiNPs-treated, and SD + SiNPs-treated groups. *Scale bar*: 50 μm. **(C)** Quantitative analysis of CD4^+^ T cell in murine ELGs, comparing the diurnal variation of positive cell ratio among the NC group, the SiNPs-treated group and the SD + SiNPs-treated group. ^**^P < 0.01. **(D)** Average abundance of CD4^+^ T cell in murine ELGs from the NC, the SiNPs-treated and the SD + SiNPs-treated groups. Statistical analysis was performed using the Kruskal–Wallis test (non-parametric), followed by Dunn’s post hoc test for multiple comparisons. ^*^P < 0.05, ^***^P < 0.001. **(E)** Quantitative analysis of CD8^+^ T cell in murine ELGs, comparing the diurnal variation of positive cell ratio among NC group, SiNPs-treated group and SD + SiNPs-treated group. For NC group, *P* = 0.3391. For SiNPs-treated group, *P* = 0.4931. For SD + SiNPs-treated group, *P* = 0.0001. ^*^P < 0.05, ^**^P < 0.01, ^***^P < 0.001. NS: not significant. **(F)** Average abundance of CD8^+^ T cells in murine ELGs from NC, SiNPs-treated and SD + SiNPs-treated groups. ^**^P < 0.01. **(G)** Heatmaps of diurnal expression for immune-related DEGs between the NC group and SiNPs-treated group in murine ELGs. The expression levels of immune-related genes were obtained from RNA-Seq and expression range of DEGs was normalized to ± 3. **(H)** The PPINs and functional clusters (cluster 1–3) with relevant KEGG pathways of immune-related genes between the SiNPs-treated group and SD + SiNPs-treated group. **(I)** The top 10 KEGG pathways enriched histogram of immune-related genes with *P* < 0.05 were displayed. **(J)** Immunoblotting of phosphorylation of STAT3, JAK2, phosphorylation of IκBα and p65, and IL17A in ELGs at ZT0 and ZT12, from NC, SiNPs-treated and SD + SiNPs-treated groups

Leukocyte migration into peripheral tissues is tightly regulated by circadian rhythms [49]. To examine the impact of SD on the immune microenvironment of the ELGs, the temporal dynamics of CD4⁺ and CD8⁺ T cell populations were analyzed over a 24-hour period. A significant increase in CD4⁺ T cell numbers was observed between the SD + SiNPs-treated group and the NC group, with SD altering the peak time of CD4⁺ T cell accumulation in the ELGs **(**Figs. 9A, C-D**)**. Similar trends were observed for CD8⁺ T cells **(**Figs. 9B, E-F**)**.

Further analysis of immune-related gene expression revealed significant transcriptional alterations in the SiNPs-treated group compared to the NC group, with SD further exacerbating these changes, as illustrated by the heatmap **(**Fig. 9G**)**. Compared to NC group, SiNPs-treated group exhibited 134 upregulated and 69 downregulated genes, while SD + SiNPs-treated group showed 81 upregulated and 122 downregulated genes (Table S16). Protein-protein interaction network (PPIN) analysis highlighted the central roles of multiple core genes within these pathways **(**Fig. 9H**)**, suggesting their potential involvement as key regulatory factors in SD- and particulate matter (PM)-induced lacrimal gland damage. Additionally, KEGG pathway enrichment analysis identified key immune pathways, including the NF-κB signaling pathway, IL-17 signaling pathway, JAK-STAT signaling pathway, chemokine signaling pathway, and Th1/Th2 cell differentiation **(**Fig. 9I and Table S17). To further evaluate the activation status of these pathways, we measured specific molecular markers: phosphorylation of STAT3 and JAK2 for the JAK-STAT pathway, phosphorylation of IκBα and p65 for the NF-κB pathway, and IL-17A expression levels for the IL-17 signaling pathway. As shown in Fig. 9J and Figures S3-S4, SiNPs significantly increased the phosphorylation of STAT3, JAK2, IκBα, and p65, as well as the expression of IL-17A. Furthermore, SD augmented these SiNPs-induced increases **(**Fig. 9J and Figures S3–S4). The dysregulation of these key immune pathways likely contributes to chronic inflammation and disruption of immune homeostasis, thereby exacerbating lacrimal gland injury and dysfunction.

Collectively, these results demonstrate that SD exacerbates SiNPs-induced immune perturbations by enhancing T cell infiltration and amplifying pro-inflammatory signaling in the lacrimal gland. The combined activation of JAK-STAT, NF-κB, and IL-17A pathways suggests a mechanism for sustained immune activation and tissue injury, contributing to the progression of dry eye pathology.

### 3.9 Neuro-related transcriptome profile alterations in the ELGs triggered by sleep deprivation in SiNPs-treated mice

**Fig. 10:**
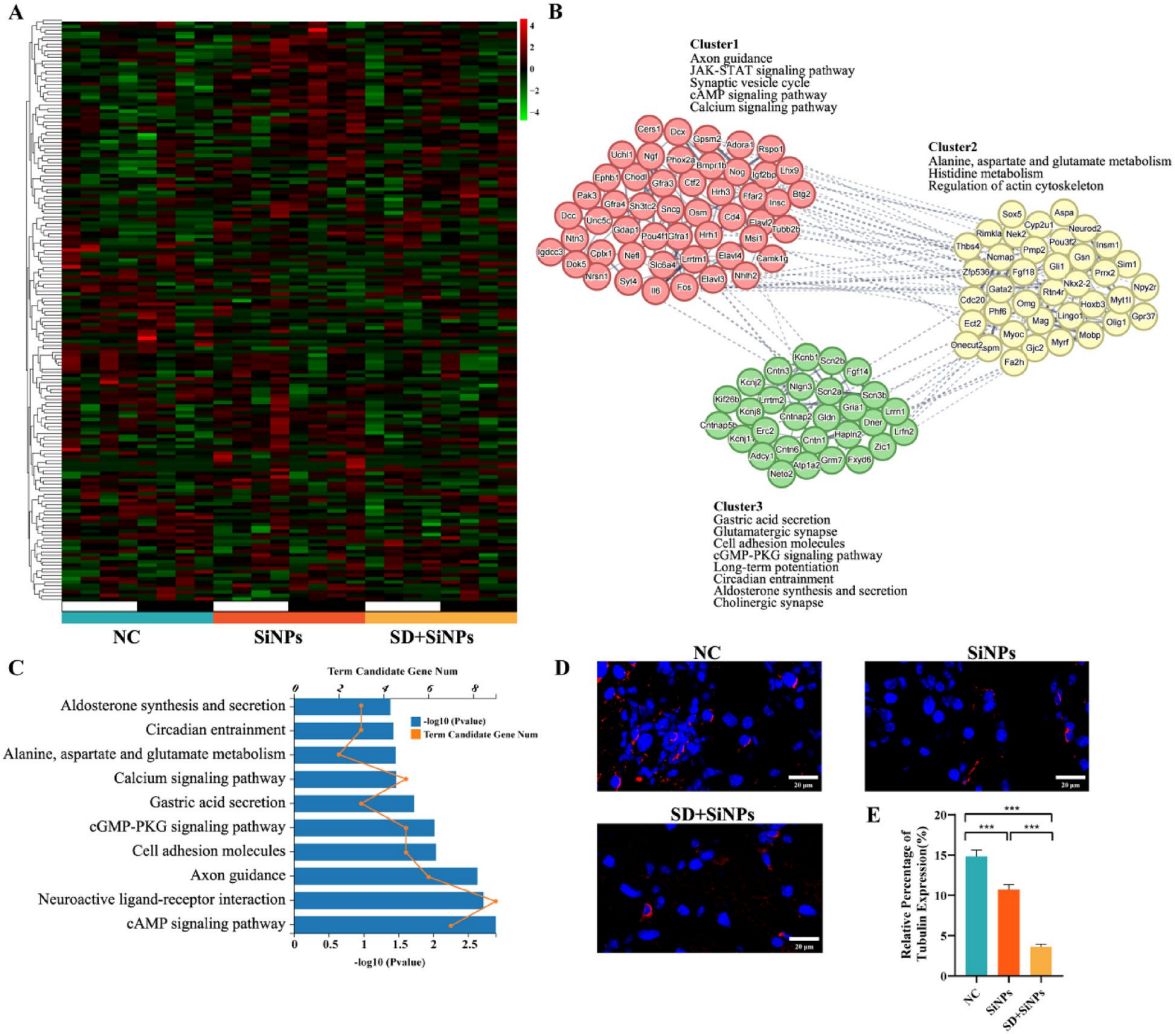
Immune alterations in murine ELGs following SiNPs and SD + SiNPs treatments. **(A)** Heatmaps of diurnal expression for nerve-related DEGs between the NC group and SiNPs-treated group in murine ELGs. The expression levels of immune-related genes were obtained from RNA-Seq and expression range of DEGs was normalized to ± 4. **(B)** The PPINs and functional clusters (cluster 1–3) with relevant KEGG pathways of nerve-related genes between the NC group and SiNPs-treated group. **(C)** The top 10 KEGG pathways enriched histogram of nerve-related genes with *P* < 0.05 were displayed. **(D)** Representative images of anti-β-III tubulin immunostaining in ELGs from the NC, SiNPs, and SD + SiNPs groups. *Scale bar*:20 μm. **(E)** Quantitative analysis of anti-β-III tubulin staining in ELGs from the NC, SiNPs, and SD + SiNPs groups. Statistical analysis was performed using Brown-Forsythe ANOVA. Multiple comparisons were conducted using the Games-Howell post hoc test. For NC vs. SiNPs, *P* = 0.0007. For NC vs. SD + SiNPs, *P* < 0.0001. For SiNPs vs. SD + SiNPs, *P* < 0.0001. ^***^P < 0.001

To evaluate the impact of SD and SiNPs exposure on neural functions in murine ELGs, nerve-related genes were identified and analyzed. A total of 98 upregulated and 74 downregulated DEGs were identified between the NC and SiNPs-treated groups, and 96 upregulated and 75 downregulated DEGs between the NC and SD + SiNPs-treated groups (Table S18). The heatmap illustrates distinct expression patterns of neuro-related genes among the NC, SiNPs-treated, and SD + SiNPs-treated groups within the LD cycle **(**Fig. 10A**)**. KEGG enrichment analysis identified the top 10 significantly enriched pathways, primarily associated with signal transduction, circadian rhythm synchronization, and neural regulation **(**Fig. 10C and Table S19).

To further explore the interactions among these genes, PPINs were constructed, categorizing neuro-related genes into three clusters **(**Fig. 10B**)**. According to KEGG functional annotation, SiNPs exposure may induce neurological dysfunction in ELGs, characterized by disruptions in signal transduction pathways, inhibition of neurogenesis, and neurotransmitter cycle disorders.

Immunofluorescence analysis of β-III tubulin, a neuronal marker, revealed decreased signal intensity in the ELGs of the SiNPs-treated and SD + SiNPs-treated mice, indicating reduced innervation or neurodegeneration (Fig. 10D). Quantification showed a significant reduction in the β-III tubulin-positive density in the SiNPs-treated group compared to the NC group, and was further diminished under SD (Fig. 10E). These findings suggest that SiNPs compromise the neural density of the ELGs in mice, potentially contributing to decreased tear secretion. SD intensifies SiNPs-induced neural damage, thereby promoting lacrimal dysfunction and dry eye progression.

### **3.10 ROS accumulation and DNA damage in the ELGs triggered by sleep deprivation in SiNPs-treated mice**

**Fig. 11:**
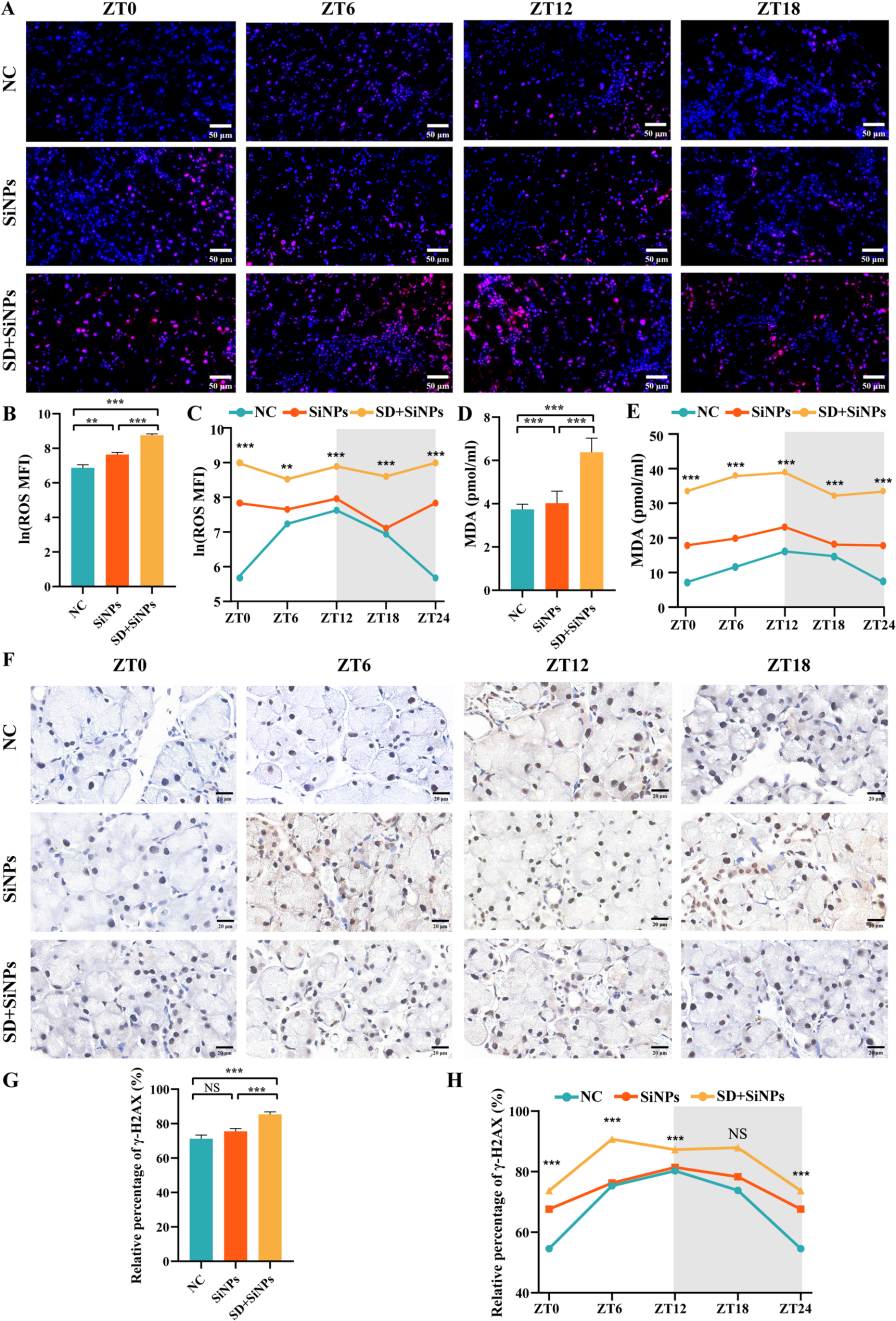
Oxidative stress and DNA damage in murine ELGs following SiNPs and SD + SiNPs treatments. **(A)** Representative images showing ROS levels at ZT0, ZT6, ZT12, and ZT18 in murine ELGs from the NC, SiNPs-treated, and SD + SiNPs-treated groups. *Scale bar*: 50 μm. **(B)** Quantitative analysis of ROS levels in murine ELGs from NC, SiNPs-treated, and SD + SiNPs-treated groups. Mean fluorescence intensity (MFI) values of ROS were log-transformed using the natural logarithm (ln) to improve normality before statistical analysis. Statistical analysis was performed using Brown-Forsythe ANOVA. Multiple comparisons were conducted using the Games-Howell post hoc test. For NC vs. SiNPs, *P* = 0.002. For NC vs. SD + SiNPs, *P* < 0.0001. For SiNPs vs. SD + SiNPs, *P* < 0.0001. ^**^P < 0.01, ^***^P < 0.001. (**C**) Diurnal changes of ROS levels in murine ELGs from NC, SiNPs-treated, and SD + SiNPs-treated groups. Statistical analyses were performed on natural log-transformed data. For the NC group, *F* = 19.65, *P* < 0.0001. For the SiNPs-treated group, *F* = 3.794, *P* = 0.0265. For the SD + SiNPs-treated group, *F* = 1.957, *P* = 0.1499. ^**^P < 0.01, ^***^P < 0.001. **(D)** Quantitative analysis of MDA levels in murine ELGs from the NC, SiNPs-treated, and SD + SiNPs-treated groups. Statistical analysis was performed using Brown-Forsythe ANOVA. Multiple comparisons were conducted using the Games-Howell post hoc test. For NC vs. SiNPs, *P* < 0.001. For NC vs. SD + SiNPs, *P* < 0.001. For SiNPs vs. SD + SiNPs, *P* < 0.001. ^***^P < 0.001. (**E**) Diurnal changes of MDA levels in murine ELGs from the NC, SiNPs, SD + SiNPs. Statistical analyses were performed on natural log-transformed data. For the NC group, *F* = 57.601, *P* < 0.001. For the SiNPs-treated group, *F* = 4.732, *P* = 0.012. For the SD + SiNPs-treated group, *F* = 2.559, *P* = 0.084. ^***^P < 0.001. **(F)** Representative images showing γ-H2AX levels at ZT0, ZT6, ZT12, and ZT18 in murine ELGs from the NC, SiNPs-treated, and SD + SiNPs-treated groups. *Scale bar*: 20 μm. **(G)** Quantitative analysis of γ-H2AX levels in murine ELGs from the NC, SiNPs-treated, and SD + SiNPs-treated groups. Statistical analysis was performed using the Kruskal–Wallis test (non-parametric), followed by Dunn’s post hoc test for multiple comparisons. For NC vs. SiNPs, *P* = 0.8374. For NC vs. SD + SiNPs, *P* < 0.0001. For SiNPs vs. SD + SiNPs, *P* = 0.0006. ^***^P < 0.001. NS: not significant. (**H**) Diurnal changes of γ-H2AX levels in murine ELGs from the NC, SiNPs-treated, and SD + SiNPs-treated groups. For the NC group, *F* = 32.35, *P* < 0.0001. For the SiNPs-treated group, *F* = 7.167, *P* = 0.0017. For the SD + SiNPs-treated group, *F* = 20.02, *P* < 0.0001. ^***^P < 0.001. NS, not significant

To examine the impact of SD on oxidative stress in the ELGs, immunofluorescence staining for ROS was performed. Compared to the NC group, the SiNPs-treated group exhibited a significant increase in ROS accumulation (*P* < 0.01). This increase was further amplified by SD, as evidenced by enhanced fluorescence intensity **(**Figs. 11A-C**)**. We also measured MDA levels, a marker of lipid peroxidation, in the ELGs. Consistent with the ROS findings, MDA levels were significantly elevated in the SiNPs group compared to the NC group (*P* < 0.001). SD further augmented this increase in MDA **(**Figs. 11D-E**)**. Furthermore, both SiNPs exposure and the combined SiNPs + SD treatment significantly disrupted oscillatory rhythms compared with the NC group **(**Figs. 11C and E**)**.

To further evaluate the impact of oxidative stress on DNA damage, immunohistochemical staining for γ-H2AX, a marker of DNA double-strand breaks, was conducted. The SD + SiNPs-treated group exhibited significantly higher γ-H2AX-positive staining compared to the NC group, indicating that ROS accumulation contributed to DNA damage in the ELGs **(**Figs. 11F-G**)**. Moreover, the oscillatory rhythm of γ-H2AX levels was disrupted by SiNPs treatment and totally changed by SD + SiNPs treatment **(**Fig. 11H**)**.

Taken together, these findings demonstrate that SiNPs exposure induces oxidative stress in the lacrimal glands, characterized by increased ROS accumulation, lipid peroxidation, and DNA damage. Importantly, SD significantly amplifies this oxidative injury and disrupts circadian redox homeostasis, likely contributing to the structural and functional decline of ELGs.

### 3.11 Activation of NLRP3 inflammasome triggered by sleep deprivation in SiNPs-treated mice

**Fig. 12:**
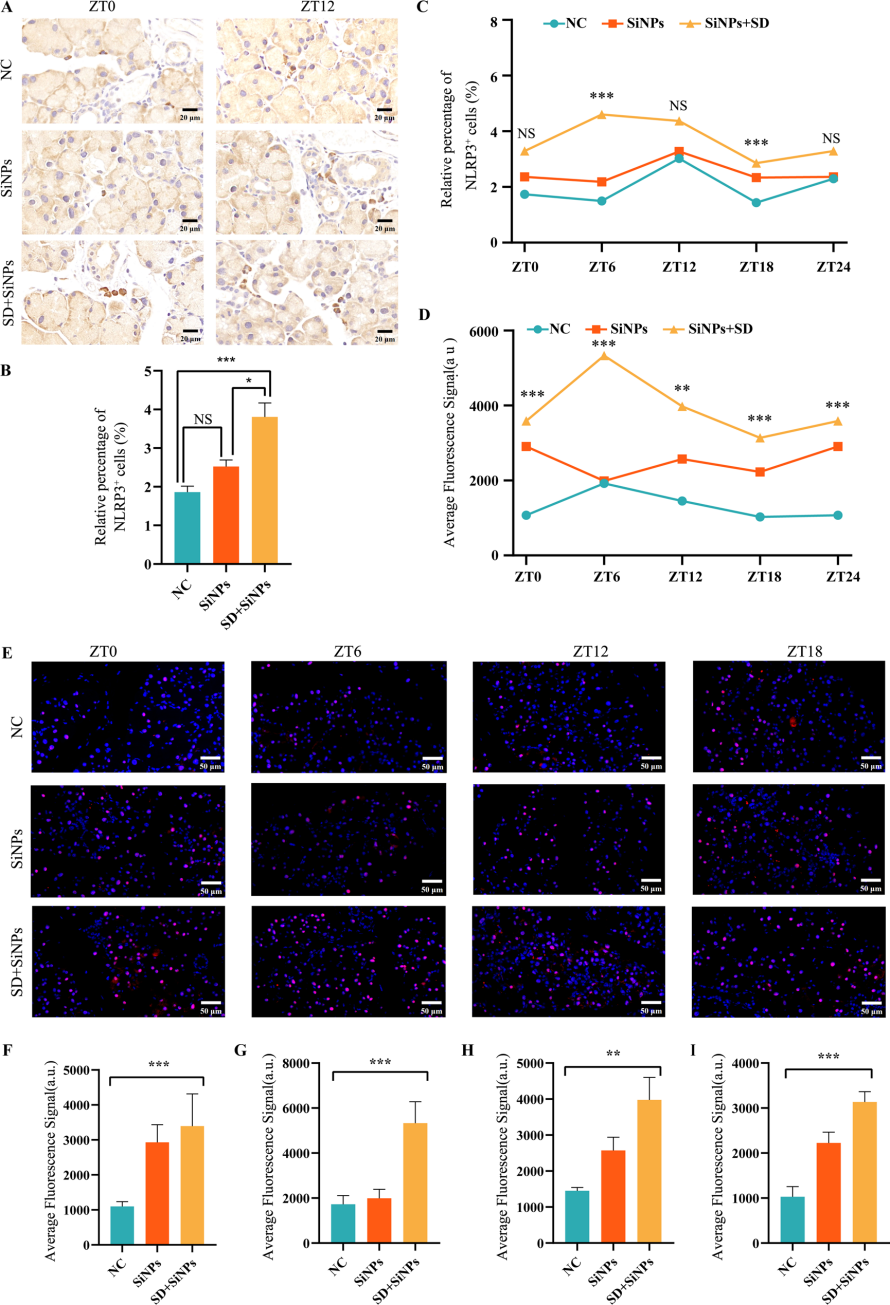
SD potentiates SiNPs-induced NLRP3 inflammasome activation in mouse ELGs. **(A)** Representative immunohistochemical images of NLRP3^+^ cells in mouse ELGs at ZT0 and ZT12 for the NC, SiNPs-treated, and SD + SiNPs-treated groups. *Scale bar*: 20 µm. **(B)** Average abundance of NLRP3^+^ cells of ELGs from NC, SiNPs-treated, and SD + SiNPs-treated groups. Statistical analysis was performed using the Kruskal–Wallis test (non-parametric), followed by Dunn’s post hoc test for multiple comparisons. Dunn’ s test showed that the SD + SiNPs-treated group differed significantly from the NC group (^***^P < 0.001) and the SiNPs-treated group (^*^P < 0.05), while the NC group, and the SiNPs-treated group were not significantly different (NS). **(C)** Diurnal variation analysis of NLRP3^+^ cell ratio in murine ELGs across the NC, SiNPs-treated group, and the SD + SiNPs-treated groups. Statistical analysis showed no significant diurnal variations in any group (ZT0: *F* = 1.419, *P* = 0.2665; ZT6: *F* = 19.05, *P* < 0.001; ZT12: *F* = 1.227, *P* = 0.3134; ZT18: *F* = 17.68, *P* < 0.001). ^***^P < 0.001. NS: not significant. **(D)** Diurnal variation analysis of ASC^+^ cells ratio in murine ELGs across NC group, SiNPs-treated group, and SD + SiNPs-treated group. For ZT0, *F* = 11.50, *P* = 0.0005; For ZT6, *F* = 10.98, *P* = 0.0008; For ZT12, *F* = 1.227, *P* = 0.005; For ZT18, *F* = 17.68, *P* < 0.0001. ^**^P < 0.01, ^***^P < 0.001. **(E)** Representative immunofluorescence images of ASC^+^ cells in mouse ELGs at ZT0, ZT6, ZT12, and ZT18 time points for the NC group, the SiNPs-treated group, and the SD + SiNPs-treated group. *Scale bar*: 50 μm. **(F-I)** Quantitative analysis of average fluorescence signal intensity of ASC in mouse ELGs at ZT0 *(G)*, ZT6 *(H)*, ZT12 *(I)*, and ZT18 *(J)* for the NC group, SiNPs-treated group, and SD + SiNPs-treated group. Statistical analysis revealed significant differences at all time points: ZT0 (*F* = 11.50, *P* = 0.0005), ZT6 (*F* = 10.98, *P* = 0.0008), ZT12 (*F* = 1.227, *P* = 0.005), and ZT18 (*F* = 17.68, *P* < 0.0001). ^**^P < 0.01, ^***^P < 0.001

The NLRP3 inflammasome plays a critical role in inflammation and oxidative stress. To investigate the involvement of the ROS/NLRP3 pathway in SiNPs-induced dry eye exacerbated by SD, we evaluated the expression patterns of NLRP3 and its adaptor protein ASC in ELGs. Immunohistochemical staining of NLRP3^+^ cells was illustrated in Fig. 12A. Analysis of NLRP3 expression revealed that SiNPs treatment alone did not significantly affect NLRP3 production in ELGs, while a marked increase was observed when SiNPs were combined with SD **(**Fig. 12B**)**, suggesting a possible synergistic effect between SiNPs treatment and SD treatment. Figure 12C showed the oscillation of NLRP3^+^ cells in the NC, the SiNPs-treated, and the SD + SiNPs-treated groups. SiNPs treatment altered the daily oscillation of NLRP3 compared to the NC group, with both groups peaking at ZT12, but exhibiting different trough values (ZT6 in the NC group vs. ZT18 in the SiNPs group). SD treatment further disrupted the daily rhythm of NLRP3, with the peak occurring at ZT6 and the trough at ZT18. Additionally, ASC levels were significantly elevated in the SiNPs-treated group compared to the NC group **(**Figs. 12E-I**)**, with its oscillatory rhythm maintained **(**Fig. 12D**)**. SD further increased ASC levels and disrupted its daily rhythmicity.

Taken together, these findings suggest that SiNPs trigger a mild activation of the NLRP3 inflammasome, which SD amplifies while deranging the rhythmic expression of NLRP3 and ASC, presumably via ROS build-up and immune imbalance.

## 4. Discussion

**Fig. 13:**
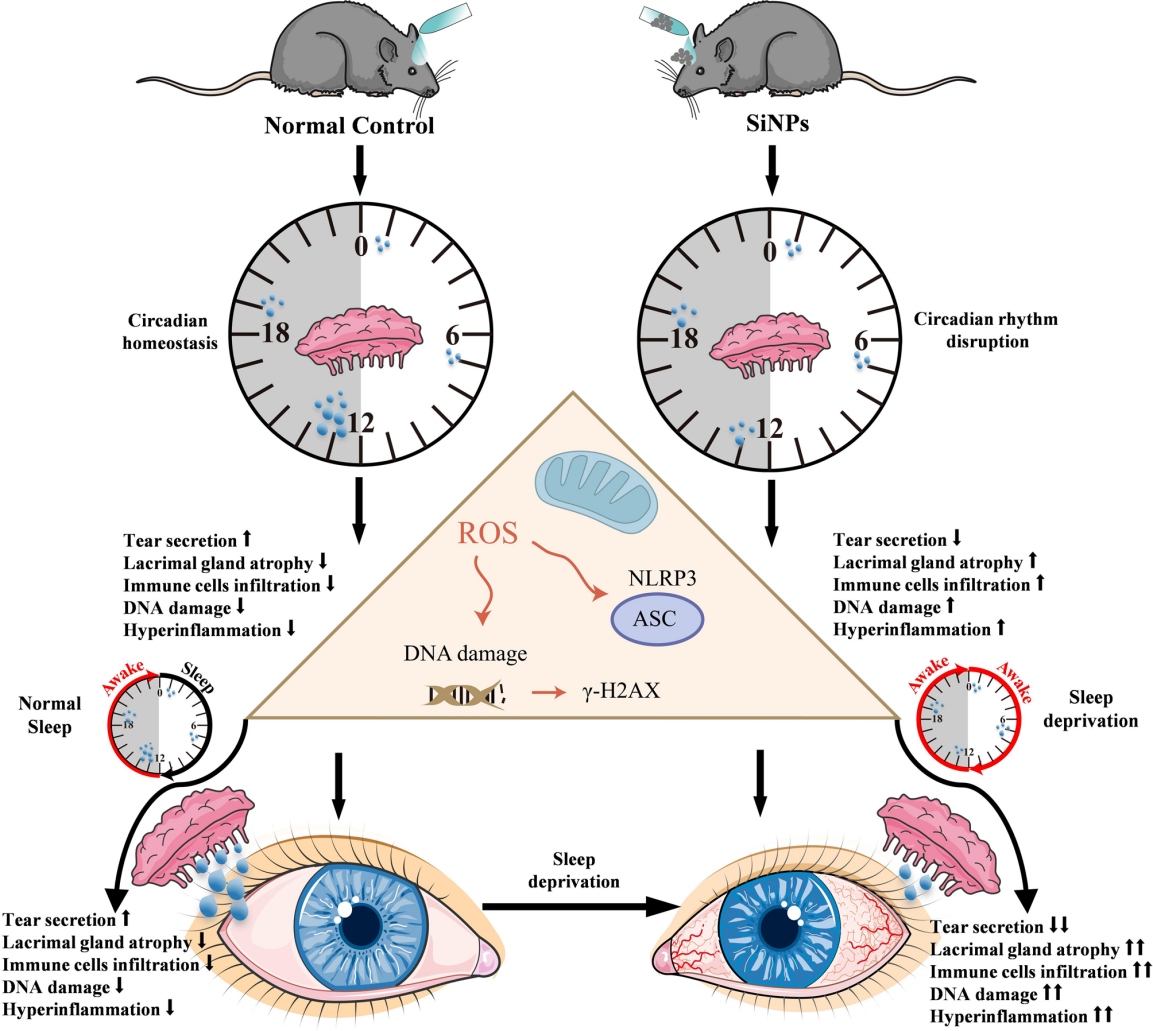
Schematic illustration of the proposed mechanism linking SiNPs exposure and sleep deprivation to dry eye pathogenesis. The diagram summarizes how combined exposure to silica nanoparticles (SiNPs) and sleep deprivation (SD) disrupts circadian homeostasis, resulting in impaired tear secretion, lacrimal gland atrophy, immune cell infiltration, and oxidative DNA damage. These pathological changes are mediated by increased reactive oxygen species (ROS) production and activation of the NLRP3 inflammasome, as indicated by elevated γ-H2AX expression. The figure underscores the interplay between circadian disruption, oxidative stress, and inflammation in driving lacrimal gland dysfunction (This figure was created using the Servier Medical ART: SMART [smart.servier.com] according to a Creative Commons Attribution 3.0 license.)

Our data show that SD magnifies the harmful impact of SiNPs on lacrimal gland function chiefly by destabilizing circadian homeostasis. We demonstrate that SD enhances SiNPs-induced behavioral arrhythmicity, transcriptomic reprogramming, immune dysregulation, oxidative stress, and structural degeneration of the ELGs. Importantly, SD potentiates oxidative damage and NLRP3 inflammasome activation via the ROS/NLRP3 signaling axis, thereby accelerating the onset and progression of dry eye disease **(**Fig. 13**)**. These findings reveal a multifaceted interaction between environmental and behavioral stressors in compromising ocular surface health. Our data not only link environmental nanotoxicity to circadian dysregulation but also uncover a mechanistic framework through which SD can exacerbate tissue injury, offering novel insights into the pathogenesis of lacrimal gland dysfunction.

The mammalian circadian system consists of a central pacemaker located in the suprachiasmatic nucleus (SCN) and peripheral oscillators located in tissues such as ELGs [50]. The peripheral clock, although synchronized by the SCN, is susceptible to environmental perturbations such as metabolic stress, photoperiodic excursions, and nanoparticle exposure [51–53]. Our previous studies also showed that ELGs, as peripheral tissues, also have circadian gene oscillations under the control of the SCN and are reprogrammed by overnutrition due to high fructose intake, streptozotocin-induced hyperglycemia, SD, and changes in light cycle phase [33, 36, 41, 48]. In the present study, we found that (1) the SiNPs and SD + SiNPs-treated groups significantly reduced ELG mass and significantly altered the pattern of circadian oscillations of ELG mass, which is consistent with the LD cycle altering the pattern of circadian oscillations of liver mass [54]; (2) the rhythmic genes of ELGs were reduced in mice after 2 weeks of SiNPs treatment (15.10% in the NC group vs. 13.50%), which is consistent with a decrease in rhythmic genes in human blood cells after sleep disruption [55, 56]; whereas SD + SiNPs treatment instead increased rhythmicity (16.26%), which suggests a different regulatory mechanism; this result is the same as the blood results of one-week sleep-deprived humans, with a significant decrease in the number of circadian-expressed genes and circadian rhythm amplitude [57]; (3) compared with the NC group, the phase of unique rhythmic genes in the SiNPs-treated group was advanced by approximately 3 h and altered the phase of rhythmic genes and the diversity of KEGG pathways; and (4) SiNPs and SD treatments altered cluster-dependent transcriptome profiles and rhythmic transcription-related KEGG pathways in ELGs. These changes explain the adverse effects of SiNPs and SD on tear function transcript levels.

SiNPs, one of the most widely produced and utilized engineered nanomaterials, have gained attention due to environmental health and safety (EHS) concerns because of increasing hazards to the environment and human exposure. Nanoparticles have been shown to inhibit angiogenesis and impair heart formation, development, and impaired cardiac function [53, 58]. Silicon dioxide nanoparticles induce iron death and liver damage in hepatocytes via ferritin autophagy [59]. SD and SiNPs synergistically reduced ELG mass, acinar cell density, and tear secretion amplitude. Histopathological analysis revealed glandular atrophy and disorganized acinar morphology, consistent with oxidative and inflammatory damage observed in dry eye models [46]. Tear secretion rhythms, which peaked at ZT12 in controls, were abolished in treated groups, paralleling disrupted locomotor activity. Notably, ELGs weight loss correlated with diminished pellet intake and body weight, suggesting systemic metabolic stress. These findings are in line with reports that circadian disruption promotes tissue atrophy in the liver, spleen, and muscle [60–62], highlighting the integral role of clock-regulated cellular maintenance in tissue integrity. The convergence of nanoparticle-induced cyto-toxicity and behavioral circadian misalignment likely impairs cellular resilience in the lacrimal gland, thereby accelerating structural and functional deterioration.

Previous research has shown that PM exerts harmful effects on the ocular surface by triggering inflammation and promoting lacrimal cell apoptosis [63, 64]. For instance, air pollutants have been reported to disrupt tear film stability, exacerbate ocular surface inflammation, and contribute to the development of dry eye disease [35, 40, 65]. SD amplified SiNPs-induced immune infiltration, with CD4^+^ and CD8^+^ T cell recruitment peaking at ZT12. Enrichment of NF-κB, IL-17, and JAK-STAT pathways underscores chronic inflammation as a key mediator of glandular dysfunction. The NLRP3 inflammasome, activated via ROS accumulation, exhibited elevated ASC levels and arrhythmic expression in the SD + SiNPs-treated ELGs, mirroring findings in neurodegenerative and metabolic disorders [66, 67]. Mechanistically, SD is known to impair immune cell trafficking by disrupting chemokine gradients and endothelial adhesion dynamics [68], a process that likely facilitated the observed lymphoid infiltration into ELG tissue. This pro-inflammatory microenvironment not only compromises glandular architecture but also exacerbates oxidative stress, forming a self-reinforcing loop between inflammation and redox damage that accelerates tissue degeneration.

Lacrimal gland secretion is tightly regulated by autonomic innervation, particularly parasympathetic inputs [69]. SD + SiNPs treatment altered synaptic signaling pathways (e.g., dopaminergic and cholinergic synapses), paralleling central nervous system synaptic remodeling observed in sleep-deprived models [36]. Reduced nerve density and aberrant neurotrophin signaling in treated ELGs suggest impaired neural control of secretion. Synaptic plasticity, known to follow circadian rhythms [36], may be disrupted by SD, uncoupling neural activity from secretory demands. These neuroanatomical and transcriptional alterations provide a mechanistic basis linking neural dysregulation to lacrimal hypofunction and offer a complementary explanation for the reduced tear secretion observed in SD + SiNPs-treated mice.

Despite profound downstream effects, the core clock machinery (*Bmal1*, *Clock*, and *Per1-3*) retained rhythmicity, albeit with phase shifts. This stability mirrors resilience observed in models of metabolic stress and aging [70, 71], where core oscillators persist despite environmental insults. However, decoupling of CCGs from core rhythms-evidenced by divergent KEGG pathway phases-suggests transcriptional or epigenetic dysregulation downstream of the core loop. Such decoupling may underlie the observed oxidative and inflammatory phenotypes, as CCGs govern redox homeostasis and immune responses [72–74]. While these findings are consistent with the concept of circadian uncoupling, the evidence remains correlative. Definitive validation will require integrative epigenomic analyses, including chromatin accessibility (e.g., ATAC-seq), DNA methylation, and histone modification profiling, to delineate the regulatory mechanisms underlying clock-output disruption in the context of SD and nanoparticle exposure.

SD combined with SiNPs exposure markedly increased ROS production and γ-H2AX accumulation in the ELG, indicating oxidative DNA damage. The accompanying loss of ROS rhythmicity is consistent with the established role of circadian clocks in redox regulation through antioxidant pathways such as NRF2/NR1D1 [75] and NRF2/BMAL1 [28, 76]. Prolonged circadian misalignment may therefore weaken endogenous antioxidant defenses, intensifying oxidative injury. Although our transcriptomic data point to a pronounced local redox imbalance within the ELG, they do not demonstrate systemic metabolic dysfunction. Vaccaro et al. [25] similarly showed that SD elevates ROS in discrete organs (e.g., the gut), supporting a model of tissue-specific vulnerability rather than a global systemic effect. Likewise, studies in the brain have linked clock-gene disruption to redox imbalance and neurodegeneration [74], but extending these mechanisms beyond the ELG requires caution. To clarify any wider ramifications, future work should include serum metabolomics, mitochondrial-respiration assays, and multi-organ redox profiling. Such studies will determine whether the oxidative stress we observe is reversible and whether systemic markers of mitochondrial dysfunction—ATP output, NAD⁺/NADH ratios, or respiratory-complex activities [73]—are altered by the combined burden of SD and nanoparticle exposure.

To further substantiate the activation of inflammatory and oxidative stress pathways revealed by transcriptomic and KEGG analyses, we performed targeted protein-level and functional assays. Western blotting confirmed increases in the phosphorylation of JAK2, STAT3, NF-κB p65, and IκBα in the SD + SiNPs-treated group, validating the engagement of both JAK-STAT and canonical NF-κB signaling pathways. Additionally, protein expression of IL-17A, a key effector in the IL-17 signaling cascade, was markedly upregulated in the combined exposure group, aligning with KEGG pathway enrichment. To assess oxidative damage functionally, ELISA for MDA showed significantly elevated levels in the SD + SiNPs-treated group, consistent with enhanced lipid peroxidation and redox imbalance. Protein and biochemical assays show that SD augments SiNPs-induced lacrimal-gland injury by jointly activating inflammatory and oxidative pathways. While our data confirm the occurrence of oxidative damage, they do not yet address the status of endogenous antioxidant defenses. Future studies should include assessment of compensatory antioxidant systems—such as SOD1, SOD2, or glutathione peroxidases—at both the protein expression and enzymatic activity levels, to gain a more complete understanding of redox dynamics in this model.

### Study limitations

Several limitations of this study should be acknowledged. While we focused on the impact of SD on the ELGs, corneal homeostasis—including nerve function and sensory feedback—was not evaluated. Given that SD may prolong ocular surface exposure and impair blink reflexes, both local inflammation and systemic effects could alter corneal sensitivity, thereby affecting lacrimal gland function via neural reflex pathways [77]. Future studies incorporating corneal assessments or alternative SD models (e.g., tarsorrhaphy) are warranted to provide a more holistic under-standing of ocular consequences. Second, the use of nocturnal C57BL/6J mice, which lack endogenous melatonin, limits the translational relevance of our findings. The inverse circadian patterns between nocturnal rodents and diurnal humans [78], combined with the absence of melatonin—a key regulator of circadian and redox homeostasis [79] —may lead to underestimation or altered interpretation of SD- and SiNPs-induced effects. Future work should employ melatonin-proficient or diurnal rodent models to better reflect human physiology. Third, our molecular analyses were restricted to transcriptomic profiling, leaving post-transcriptional and metabolic mechanisms unexplored. Integrating proteomics and metabolomics in future studies would provide a more comprehensive view of the molecular landscape underlying SD- and SiNPs-induced ELG dysfunction [80]. Fourth, variability in SD induction—including timing, duration, and implementation method—may introduce experimental confounders. Standardization of SD protocols will be essential in future research to delineate the specific contributions of sleep loss to lacrimal gland pathology [70]. Finally, while SiNPs served as a surrogate for environmental PM, they do not fully replicate the complexity of real-world air pollution, which includes a heterogeneous mixture of ultrafine and fine PM with potential synergistic toxicities [81]. Future studies should evaluate the impact of authentic PM exposure under environmentally relevant conditions to enhance ecological validity and translational applicability.

## Conclusion

This study demonstrates that SD significantly exacerbates silica nanoparticle-induced toxicity in lacrimal glands by disrupting circadian rhythms and enhancing oxidative stress and inflammation. In addition to transcriptomic alterations, we validated key pro-inflammatory and oxidative pathways at the protein level, including activation of the JAK-STAT and NF-κB signaling cascades and upregulation of IL-17A. Elevated MDA levels confirmed redox imbalance and lipid peroxidation in the lacrimal gland. Although the expression of antioxidant enzymes was not directly assessed, the collective evidence reveals a mechanistic basis for SD-amplified nanotoxicity through ROS/NLRP3-mediated immune activation. These findings highlight the importance of accounting for both behavioral and environmental risk factors in the pathogenesis of ocular surface diseases, such as dry eye. Future studies employing melatonin-proficient or diurnal animal models, as well as therapeutic interventions targeting the ROS/NLRP3/IL-17A axis, may provide promising strategies for preventing or mitigating dry eye disease induced by circadian disruption and environmental particulate exposure.

## Supplementary Information

Table S1

Table S2

Table S3

Table S4

Table S5

Table S6

Table S7

Table S8

Table S9

Table S10

Table S11

Table S12

Table S13

Table S14

Table S15

Table S16

Table S17

Table S18

Table S19

Supplementary Material

## Data Availability

Additional data related to this article are available through NCBI’s BioProject database under accession PRJNA1243066.
