# Supplementary material for: Circadian disruption and ROS-NLRP3 signaling mediate sleep deprivation-enhanced silica nanoparticle toxicity in lacrimal glands
Source: J Nanobiotechnology. 2025 Sep 2;23:600. doi: 10.1186/s12951-025-03630-5 (PMC12403269; doi:10.1186/s12951-025-03630-5)
Supplement: Supplementary file 20 — Supplementary Material [file 12951_2025_3630_MOESM20_ESM.docx]

Supplementary Information

**Title:** **Sleep Deprivation Potentiates Silica Nanoparticle-Induced Circadian Disruption and Lacrimal Gland Dysfunction in Mice**

**Short title: SD Aggravates SiNP-Induced Lacrimal Gland Dysfunction**

Wenxiao Zhang^1^, Di Qi^2^, Xiaoting Pei^2^, Dingli Lu^2^, Mengru Ba^1^, Shuting Xuan^3^, Duliurui Huang^1^, Tingting Yang^3^, Zhijie Li^1,2,3*^, Shenzhen Huang^1,2,3*^

^1^Department of Ophthalmology, People’s Hospital of Zhengzhou University, Henan Provincial People’s Hospital, Zhengzhou, China

^2^Henan Eye Institute, Henan Eye Hospital and Henan Key Laboratory of Ophthalmology and Visual Science, People’s Hospital of Henan University, People’s Hospital of Zhengzhou University, Henan Provincial People’s Hospital, Zhengzhou, China

^3^Department of Ophthalmology, People’s Hospital of Henan University, Henan Provincial People’s Hospital, Zhengzhou, China

***Corresponding author**

Henan Eye Institute & Henan Eye Hospital, Henan Provincial People’s Hospital, No. 7, Weiwu Road, Zhengzhou, China (450003), Tel: +86-371-67120562; Fax: +86-371-67120562. Email address: tzhijieli@jnu.edu.cn (Zhijie Li), huangshenzhen@zzu.edu.cn (Shenzhen Huang)

**
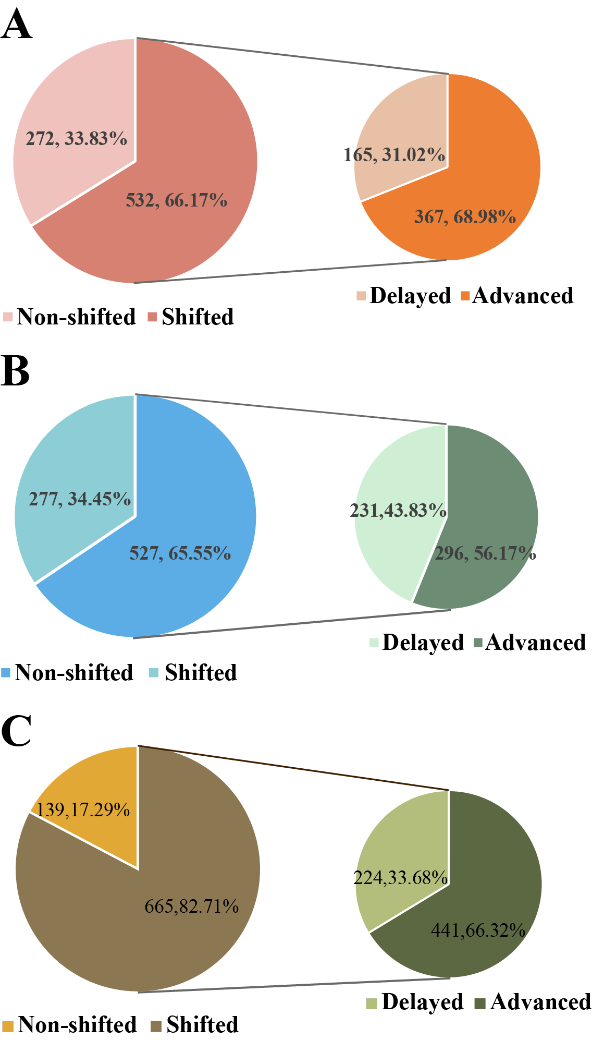
**

**Figure S1. Phase shifts of rhythmic genes in response to SiNPs and SD+SiNPs treatments.**

**(A)** Pie charts illustrate the distribution of non-shifted and shifted rhythmic genes in the SiNPs-treated group compared to the NC group. Of the 804 rhythmic genes in the NC group, 272 (33.83%) remained non-shifted, while 532 (66.17%) underwent phase shifts. Among the shifted genes, 367 (68.98%) were delayed and 165 (31.02%) were advanced.

**(B)** Pie charts show the phase shift distribution in the SD+SiNPs-treated group compared to the SiNPs-treated group. In the SD+SiNPs-treated group, 277 (34.45%) of 804 genes remained non-shifted, while 527 (65.55%) were shifted. Specifically, 296 (56.17%) were delayed and 231 (43.83%) were advanced.

**(C)** Pie charts depict the phase shifts of rhythmic genes in the NC group under SD+SiNPs treatment. Of the 804 genes, 139 (17.29%) remained non-shifted, while 665 (82.71%) were shifted. Among the shifted genes, 441 (66.32%) were advanced and 224 (33.68%) were delayed. *N*=24 individual mice per group.


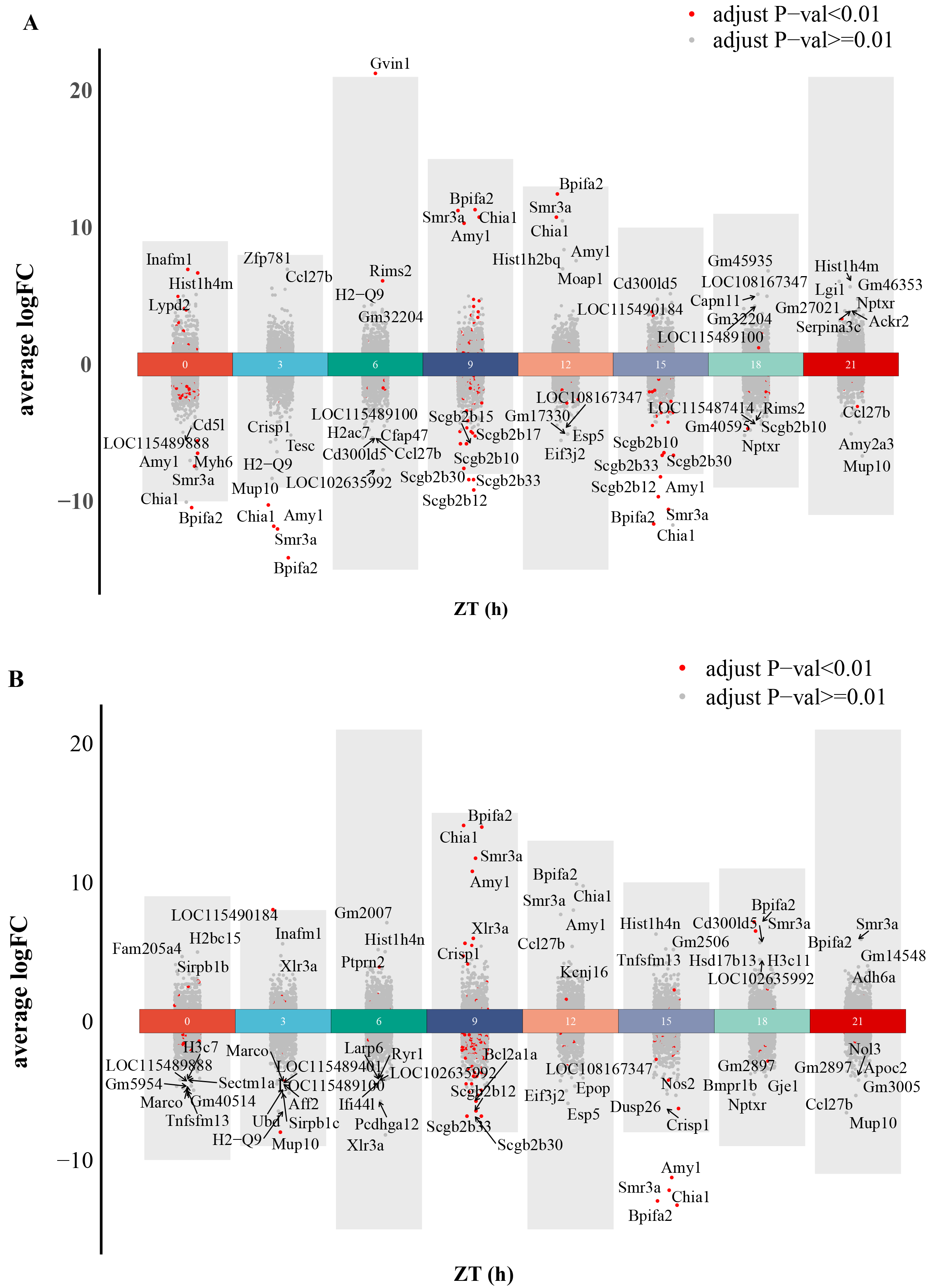


**Figure S2. Differential gene expression analysis in murine ELGs following SiNPs treatment and SD treatment.**

**(A)** Volcano plot visualizing DEGs between NC and SD+SiNPs-treated groups. The x-axis indicates ZT points, and the y-axis shows fold change (FC). Red dots represent genes with adjusted *P* < 0.01, while gray dots indicate genes with adjusted *P* ≥ 0.01. *N*=24 individual mice per group.

**(B)** Volcano plot comparing DEGs between SiNPs-treated and SD+SiNPs-treated groups. The x-axis indicates ZT points, and the y-axis shows FC. Red dots represent genes with adjusted *P* < 0.01, while gray dots indicate genes with adjusted *P* ≥ 0.01. *N*=24 individual mice per group. *N*=24 individual mice per group.
